# The BMP signaling gradient is interpreted through concentration thresholds in dorsal–ventral axial patterning

**DOI:** 10.1371/journal.pbio.3001059

**Published:** 2021-01-22

**Authors:** Hannah Greenfeld, Jerome Lin, Mary C. Mullins

**Affiliations:** 1 Department of Cell and Developmental Biology, University of Pennsylvania Perelman School of Medicine, Philadelphia, PA, United States of America; 2 Institute for Biomedical Informatics, University of Pennsylvania Perelman School of Medicine, Philadelphia, PA, United States of America; University of Edinburgh, UNITED KINGDOM

## Abstract

Bone Morphogenetic Protein (BMP) patterns the dorsal–ventral (DV) embryonic axis in all vertebrates, but it is unknown how cells along the DV axis interpret and translate the gradient of BMP signaling into differential gene activation that will give rise to distinct cell fates. To determine the mechanism of BMP morphogen interpretation in the zebrafish gastrula, we identified 57 genes that are directly activated by BMP signaling. By using Seurat analysis of single-cell RNA sequencing (scRNA-seq) data, we found that these genes are expressed in at least 3 distinct DV domains of the embryo. We distinguished between 3 models of BMP signal interpretation in which cells activate distinct gene expression through interpretation of thresholds of (1) the BMP signaling gradient slope; (2) the BMP signal duration; or (3) the level of BMP signal activation. We tested these 3 models using quantitative measurements of phosphorylated Smad5 (pSmad5) and by examining the spatial relationship between BMP signaling and activation of different target genes at single-cell resolution across the embryo. We found that BMP signaling gradient slope or BMP exposure duration did not account for the differential target gene expression domains. Instead, we show that cells respond to 3 distinct levels of BMP signaling activity to activate and position target gene expression. Together, we demonstrate that distinct pSmad5 threshold levels activate spatially distinct target genes to pattern the DV axis.

## Abstract 2

This study tested three models of how a BMP morphogen gradient is translated into differential gene activation that specifies distinct cell fates, finding that BMP signal concentration thresholds, not gradient shape or signal duration, position three distinct gene activation domains.

## Introduction

During embryonic patterning, the molecular identity of unspecified cells is determined by the location of each cell within the embryo [1]. Thereby, a cell’s position becomes translated into a specific cell fate. This positional information is provided by gradients of secreted signaling molecules called morphogens, which induce the specification of multiple cell fates [2–5]. The genetic programs underlying different cell fates are activated in distinct regions of tissue within the morphogen gradient. The proper position of gene expression boundaries is essential during development because these domains determine the differentiation and relative abundance of distinct cell types. A fundamental question in developmental biology is how a graded morphogen signal is translated into discrete gene expression domains to specify cell fate.

Multiple mechanisms have been proposed for how morphogen gradients provide positional information to pattern a tissue. Cells in different positions along the gradient may respond to (1) different signaling concentrations; (2) different lengths of signal exposure; or (3) different spatial slopes of the signaling gradient. There is evidence that cells can read out each of these aspects of a morphogen gradient to activate different genes in different contexts. For example, the Bicoid transcription factor morphogen gradient activates target genes through different concentration thresholds to pattern the anterior–posterior (AP) axis of *Drosophila* [6,7]. In contrast, the duration of Sonic Hedgehog (Shh) ligand exposure has been shown to pattern gene expression in the vertebrate neural tube and in the limb bud [8,9]. There is conflicting evidence for the mechanism of Bone Morphogenetic Protein (BMP) morphogen interpretation. Both the duration of BMP signaling [10] and different BMP ligands [11] have been suggested as mechanisms responsible for establishing dorsal interneuron identities in the neural tube, while BMP has been shown to pattern gene expression in a concentration-dependent manner in human and mouse embryonic stem cells [12,13]. While in *Drosophila*, there is evidence that cells read out the spatial slope of the Dpp (the BMP homolog) gradient to regulate cell proliferation in the imaginal wing disc [14]. Dpp signaling was shown to regulate the activity of the intercellular Fat signaling pathway, allowing cells to sense differences in signaling activity between neighboring cells [15].

A BMP morphogen signaling gradient is required early in embryonic development to pattern the dorsal–ventral (DV) embryonic axis in vertebrates and invertebrates [16–18]. Despite its fundamental role to pattern tissues along the DV axis in all metazoans, the mechanism by which the BMP signaling gradient is interpreted into positional information and multiple cell fates along the axis is not known. In the zebrafish (*Danio rerio*), loss of BMP signaling eliminates epidermis, placodes, neural crest, and ventral mesendoderm and leads to a massive expansion of neural tissues [19–21]. A gradient of BMP2/7 signaling activity across the embryo forms during gastrulation, with the highest level of signaling ventrally and the lowest levels dorsally [22,23]. This signaling gradient is transduced through a heterotetrameric receptor complex that phosphorylates and activates the transcription factor, Smad5 [24]. BMP signaling activity is thus interpreted by cells through nuclear accumulation of phosphorylated Smad5 (pSmad5) to specify different ventral cell fates [25]. pSmad5 in complex with co-Smad4, in turn, activates gene expression [26,27].

Changing the level of BMP signaling across the embryo shifts the position and relative proportion of ventral cell fates, demonstrating that the gradient shape is important for patterning [20,28–32]. The shape of the BMP signaling gradient depends on the extracellular antagonist Chordin, which binds the BMP ligand and blocks signaling in the dorsal region of the embryo [23,28,33–35]. The BMP signaling gradient is dynamic during late blastula to mid-gastrula stages; thus, pSmad5 nuclear levels differ across the embryo both in space and time [22,23]. However, it remains unknown if gradient shape or gradient dynamics play a role in positioning gene expression domains that specify ventral cell fates along the DV axis.

Here, we investigated the mechanism by which BMP signaling provides positional information to cells across the DV embryonic axis. We identified greater than 50 genes that directly read out the BMP signaling gradient to specify distinct ventral–lateral cell fates along the DV axis. We tested 3 prominent mechanisms of morphogen gradient interpretation: interpretation by signaling gradient slope, by BMP signal duration, and by signaling gradient concentration thresholds. By using quantitative measurements of pSmad5 in all nuclei of the embryo to investigate the spatial relationship between BMP signaling activity levels and the activation of different target genes, we eliminated BMP gradient slope and BMP signal duration as mechanisms positioning target gene expression. We determined that cells respond to at least 3 distinct levels of pSmad5 to activate different target genes, and these threshold levels of pSmad5 can precisely position gene expression boundaries in the embryo.

## Results

### Identification of target genes directly patterned by the BMP gradient

**Fig 1:**
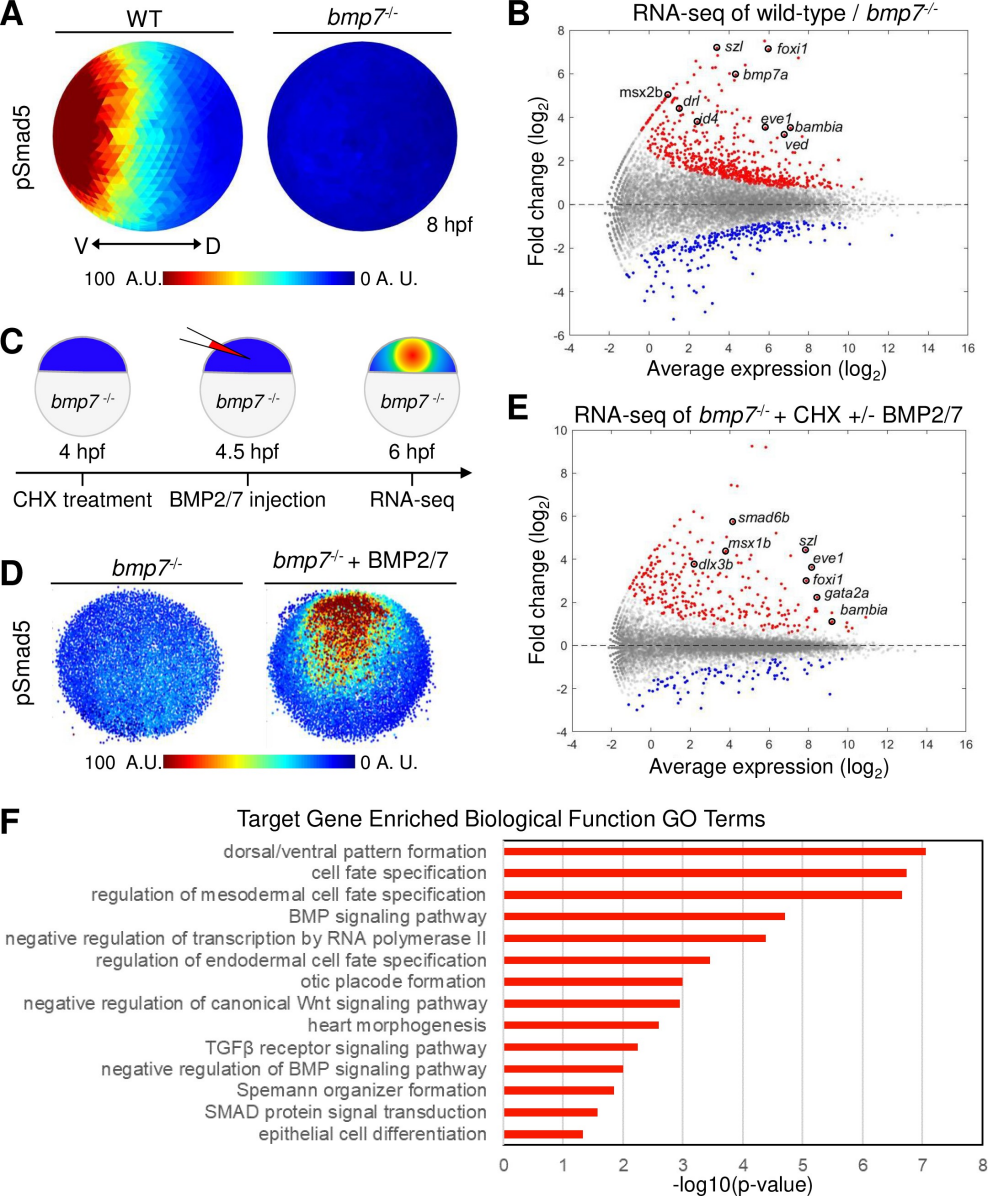
Direct targets of BMP signaling in DV axial patterning. **(A)** Animal view, dorsal to right of mean pSmad5 intensities at mid-gastrula stage (8 hpf) WT (*n* = 4) and *bmp7* mutant (*n* = 3) embryos. **(B)** Differential gene expression in WT and *bmp7* mutants at 8 hpf using RNA-seq. Significantly up-regulated genes in WT compared to *bmp7* mutants are shown in red, and significantly down-regulated genes are shown in blue. All other genes are shown in gray. A subset of known BMP-dependent genes is highlighted. See Table A in S1 Data for underlying data. **(C)** Schematic of method to isolate RNA for sequencing from CHX-treated *bmp7* mutant embryos injected with BMP2/7 protein. **(D)** Representative image of pSmad5 intensities of all nuclei of an individual *bmp7* mutant (*n* = 9) and *bmp7* mutant injected with 10 pg of BMP2/7 protein (*n* = 9). Animal pole facing up. **(E)** Differential gene expression using RNA-seq of CHX-treated *bmp7* mutants versus CHX-treated *bmp7* mutants injected with BMP2/7 protein. Significantly up-regulated genes with BMP2/7 protein injection are shown in red, and significantly down-regulated genes are shown in blue. All other genes are shown in gray. A subset of BMP direct target genes is highlighted. See Table B in S1 Data for underlying data. **(F)** GO term analysis for biological processes of the 57 direct target genes. See Table C in S1 Data for underlying data. A.U. is arbitrary units. BMP, Bone Morphogenetic Protein; CHX, cycloheximide; DV, dorsal–ventral; GO, Gene Ontology; hpf, hours post fertilization; pSmad5, phosphorylated Smad5; RNA-seq, RNA sequencing; TGFβ, Transforming Growth Factor Beta; WT, wild-type.

BMP signaling is essential for ventral tissue specification, but it remains unknown how graded BMP signaling is interpreted by cells of the embryo to generate the distinct gene expression domains that pattern the DV axis. Only a limited number of genes directly regulated by BMP signaling have been identified in the zebrafish gastrula. To identify the genes responding to the BMP gradient, we determined which genes are directly activated by pSmad5 from all BMP-dependent gene expression during gastrulation. BMP-dependent gene expression was determined by performing RNA sequencing (RNA-seq) on wild-type and *bmp7* mutant embryos (*bmp7a*^*sb1aub*^) at 2 time points when the BMP signaling gradient patterns ventral tissues: early (shield) and mid-gastrula (70% epiboly) stages [22,36,37]. All ventral tissue specification is absent in *bmp7* mutants, and the activation of Smad5 is abolished (Fig 1A, S1A and S1B Fig) [20,21,38]. We identified 1,559 genes that were differentially expressed (false discovery rate [FDR] < 0.05) in *bmp7* mutant compared to wild-type embryos at an early gastrula stage (S1C Fig) and 852 genes that were differentially expressed at mid-gastrulation (Fig 1B). Most differentially expressed genes were down-regulated in *bmp7* mutants compared to wild-type embryos at both early and mid-gastrula stages, including many known markers of ventral tissues reflecting a loss of ventral cell fates (Fig 1B, S1C Fig).

To identify the subset of BMP-dependent genes that are directly regulated by BMP signaling, we treated *bmp7* mutant embryos at 4 hours post fertilization (hpf) with cycloheximide (CHX), a translation inhibitor, and then injected BMP2/7 recombinant protein into the intercellular space of the blastula (Fig 1C). First, we chose 4 hpf because the zygotic genome has been activated by this time point [39–41], but the DV axis has yet to be patterned, and all cells of the embryo remain competent to respond to BMP signaling [22]. Second, translation was inhibited with CHX to prevent the expression of secondary targets but not direct targets of BMP signaling. Finally, embryos were injected with BMP2/7 protein to activate BMP signaling and induce robust phosphorylation of Smad5 in *bmp7* mutant embryos (Fig 1D).

Total RNA was isolated 1.5 hours postinjection from *bmp7* mutant embryos treated with CHX with or without BMP2/7 protein injection for RNA-seq analysis. We identified 363 genes that were differentially expressed (FDR < 0.05) in embryos injected with BMP2/7 protein (Fig 1E). In BMP2/7 injected and CHX-treated embryos, a known direct target of BMP signaling, *foxi1* [42], was confirmed to be expressed by in situ hybridization 1.5 hours after injection (S1D Fig). We compared the 274 genes that were up-regulated by BMP signaling after CHX treatment to the genes that were BMP-dependent during gastrulation. We found 57 genes that are both directly up-regulated by BMP signaling and endogenously expressed during gastrulation when ventral tissues are specified (S1 Table). Gene Ontology (GO) analysis of the 57 target genes found enrichment of terms for cell fate specification and tissue differentiation (Fig 1F). The BMP target genes were also enriched for GO terms for DNA-binding transcription factor activity (S1E Fig), which together are consistent with roles in specifying ventral cell fates. Fifteen genes were found to be both down-regulated by BMP signaling after CHX treatment and down-regulated in wild-type embryos compared to *bmp7* mutants indicating that pSmad5 can directly inhibit gene expression (S2 Table). There is evidence that BMP-activated Smad transcription factors directly repress transcription via recruitment of repressors and chromatin modifiers [43–45]. Ten of these down-regulated target genes are known to be expressed within dorsal ectodermal tissue, which is consistent with a role in direct down-regulation by BMP signaling [23,46–54]. Thus, we identified genes that directly read out the BMP signaling gradient to initiate the genetic cascade that specifies different ventral cell fates [37,42,55–57]. We now have a comprehensive list of genes directly responding to the BMP gradient during DV patterning.

### Ventral BMP target genes are expressed in at least 3 distinct domains

**Fig 2:**
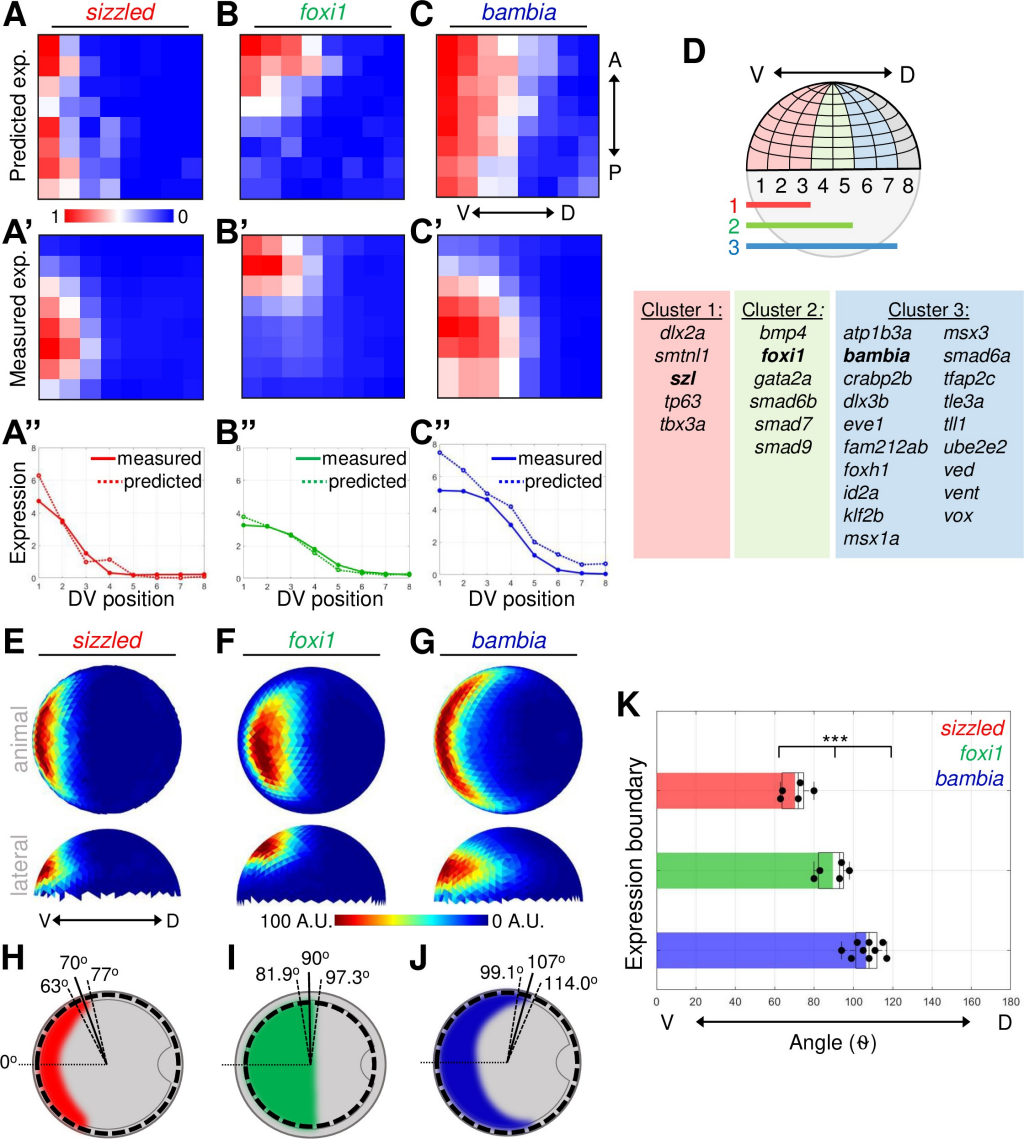
Three distinct expression domains of BMP direct target genes. **(A–C)** Heat map of gene expression patterns from Seurat analysis of mid-gastrula (8 hpf) scRNA-seq dataset. Predicted expression normalized across all bins of *sizzled* **(A)**, *foxi1* **(B)**, and *bambia* **(C)**. **(A’–C’)** Average FISH intensity measured in early gastrula (7 hpf) WT embryos divided into 64 equally spaced bins. **(A”–C”)** Measured and Seurat predicted expression profiles across the DV axis. Each point is the sum of the expression intensity from all bins at 1 DV position. See Tables A–C in S2 Data for underlying data. **(D)** Schematic of embryonic grid divided into 64 bins and the 3 nested expression domains. Table of 27 ventrally expressed target genes divided into 3 clusters based on their Seurat expression profiles. **(E–G)** Animal and lateral views of average FISH signal in WT embryos at early gastrula (7 hpf) of *sizzled* (*n* = 5) **(E)**, *foxi1* (*n* = 5) **(F)**, and *bambia* (*n* = 9) **(G)**. **(H–J)** Schematic of animal view (dorsal to right) of expression domains in early gastrula embryos of *sizzled* **(H)**, *foxi1* **(I)**, and *bambia* **(J)**. The mean (solid line) and standard deviation (dotted lines) of expression boundaries shown as degrees across the DV axis. Position of domain boundary measured from the average intensity from a 40-μm band of cells across the DV axis at the location indicated by the dotted circle. **(K)** DV position of the expression boundaries of individual WT embryos. See Table K in S2 Data for underlying data. ***P < 0.001 in comparing 3 expression domains using 1-way ANOVA. A.U. is arbitrary units. ANOVA, analysis of variance; BMP, Bone Morphogenetic Protein; DV, dorsal–ventral; FISH, fluorescent in situ hybridization; hpf, hours post fertilization; scRNA-seq, single-cell RNA sequencing; WT, wild-type.

Next, we determined where the genes reading out the BMP signaling gradient are expressed along the DV axis of the embryo. Target genes responding to different aspects of the gradient would be predicted to be expressed in different domains of the embryo. To sort the target genes based on their expression pattern, we analyzed a previously published single-cell RNA sequencing (scRNA-seq) dataset of mid-gastrula zebrafish embryos using the Seurat method, which predicts the spatial position of individual cell transcriptomes [58,59]. Locations of single-cell transcriptomes were inferred by Seurat based on the co-expression of known landmark genes and mapped onto an embryonic grid that was divided into 64 bins: 8 bins across the DV axis and 8 bins across the animal–vegetal (AV) axis (Fig 2D). The predicted expression patterns of the BMP direct target genes within the 64 bins across the embryo are shown as a heat map (Fig 2A–2D, S2–S5 Figs). To visualize the predicted expression profiles across the DV axis, we summed expression in all bins within each of the 8 positions along the DV axis (Fig 2A”–2C”, S2–S5 Figs).

Genes were then clustered based on their predicted expression profiles across the DV axis. Fifty of the 57 up-regulated target genes were found in the scRNA-seq dataset, and 27 genes showed predicted ventrally restricted expression (S2–S4 Figs). These ventrally restricted target genes were further sorted based on the bins where they were predicted to be expressed across the DV axis. Five genes had predicted expression enriched within the first 3 to 4 bins, 6 genes were predicted to be expressed within the first 5 bins, and 19 genes within the first 6 to 7 bins (Fig 2D, S2–S4 Figs). Target genes with uniform expression or that also included dorsal expression were assumed to have multiple signaling inputs and excluded from our analysis (S5 Fig, S1 Table). The down-regulated target genes that were sequenced in the scRNA-seq dataset displayed either dorsal enrichment or were not enriched along the DV axis (S6 Fig, S2 Table). To further investigate how the pSmad5 gradient is interpreted into spatially distinct gene expression domains, we focused on genes that are induced exclusively by BMP signaling. Some of the BMP target genes are known to have multiple signaling pathways contributing to the total expression pattern. For example, the expression of *eve1* is known to be activated by both BMP as well as by Fibroblast Growth Factor (FGF) signaling [60–62]. Similarly, we also avoided examining genes that have significant expression remaining in *bmp7* mutant embryos (S1 Table).

For representative BMP targets, we chose 1 gene from each cluster to investigate how the gradient of BMP signaling directs target gene expression: *sizzled* in cluster 1, *foxi1* in cluster 2, and *bambia* in cluster 3. To validate the predicted expression patterns of the 3 target genes, we performed fluorescent in situ hybridization (FISH) for each gene on early gastrula (7 hpf) wild-type embryos (S7A Fig), when the BMP gradient is patterning ventral fates [22,37]. Each embryo was subdivided into 128 bins equally spaced across the AV and DV axes (S7B Fig). Expression intensity for each lateral half of the embryo was averaged into 64 bins, normalized, and displayed similarly as the Seurat heat map (Fig 2A’–2C’). The measured and predicted expression profiles of the 3 target genes are similar across the DV axis (Fig 2A”–2C”) validating this approach for examining the DV expression domain. While Seurat accurately predicted differences in expression patterns of the 3 genes across the DV axis, *sizzled* and *bambia* were incorrectly predicted to be uniformly expressed along the AV axis. Using the quantified FISH for these target genes and other landmark genes will improve the accuracy of Seurat’s predicted expression pattern across the AV axis of the embryo.

To more precisely determine where the 3 target genes are expressed across the DV axis, we quantified the FISH expression intensity (Fig 2E–2G, S7A Fig). The expression of *sizzled*, *foxi1*, and *bambia* was quantified around the DV axis of the embryo at the AV location of highest intensity (S7C–S7E Fig). While *sizzled* and *bambia* are expressed more broadly along the AV axis, *foxi1* is a marker for nonneural ectoderm and only expressed in an animal–ventral domain of the embryo [37]. We measured target gene expression boundaries in individual embryos (S7C–S7E Fig). We found that the boundaries of the target genes are located in distinct positions along the DV axis (Fig 2H–2J), with each target gene expressed in a significantly different domain of the embryo (Fig 2K). These results together with the Seurat analysis of 27 direct target genes indicate that the BMP signaling gradient can pattern the embryo into at least 3 distinct gene expression domains.

### Distinct pSmad5 levels and gradient slopes correspond to different target gene expression boundaries

**Fig 3:**
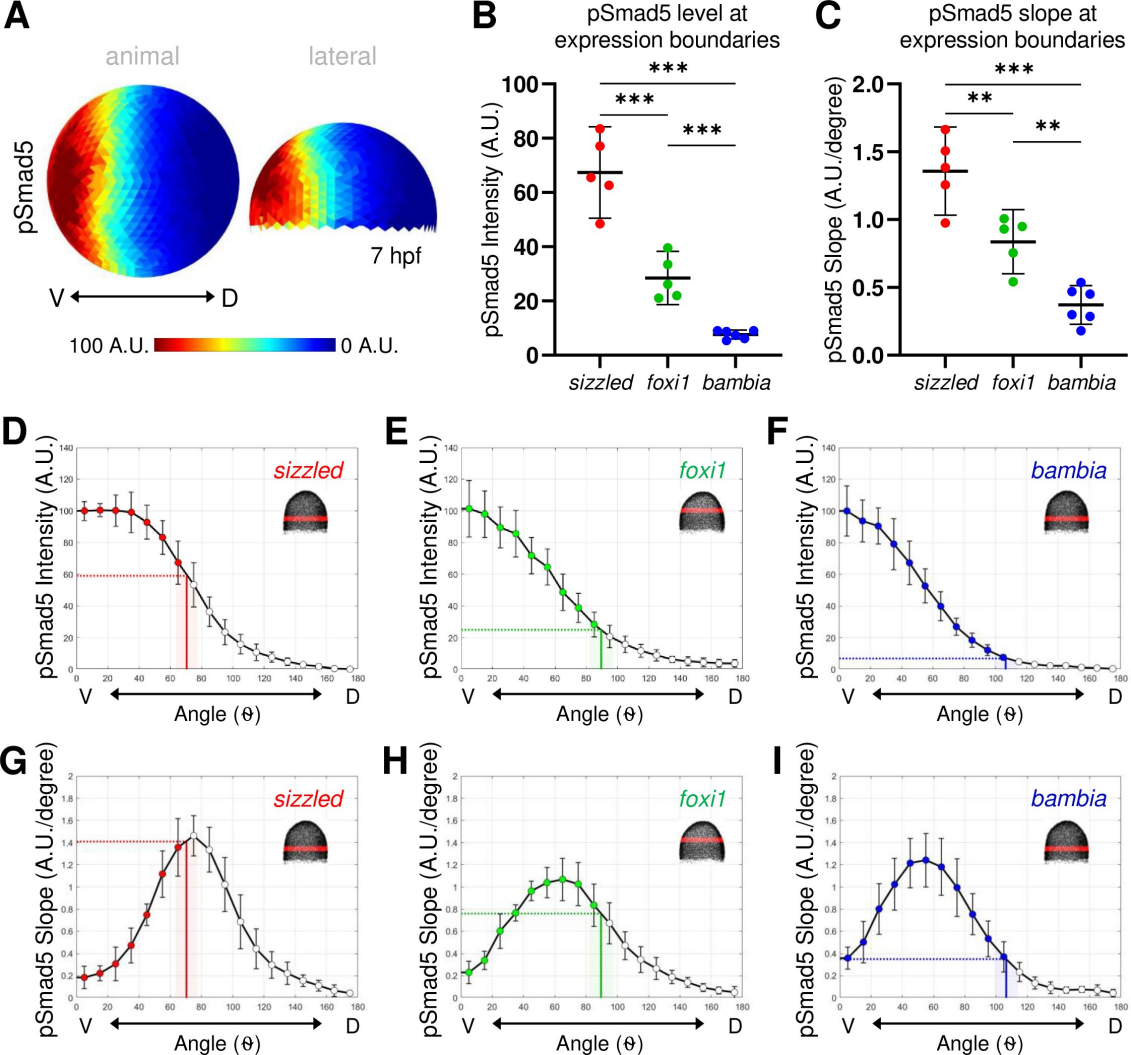
pSmad5 gradient with distinct thresholds and slopes delineating expression domains. **(A)** Animal and lateral view of average pSmad5 intensities in early gastrula (7 hpf) WT embryos (*n* = 5). **(B)** Measurement of pSmad5 intensity at the expression boundaries of *sizzled* (red), *foxi1* (green), and *bambia* (blue) across the DV axis of WT embryos at 7 hpf. See Table A in S3 Data for underlying data. **(C)** Measurement of pSmad5 gradient slope at the expression boundaries of *sizzled* (red), *foxi1* (green), and *bambia* (blue) across the DV axis of WT embryos at 7 hpf. See Table B in S3 Data for underlying data. **(D–F)** WT pSmad5 profiles across the DV axis. The intensity is averaged from a 40-μm band of cells around the DV axis at the location shown in red in the right corner embryo schematic of each panel. One WT clutch was used for **(D, E, G, H)** (*n* = 5), another WT clutch was used for **(F, I)** (*n* = 6). Positions of expression boundaries for *sizzled* **(D)**, *foxi1* **(E)**, and *bambia* **(F)** are shown as vertical solid lines. Level of pSmad5 at the boundary is indicated as a horizontal dotted line. Colored dots indicate positions where target genes are expressed. Standard deviations of expression boundaries are shaded. See Tables C–E in S3 Data for underlying data. **(G–I)** Slopes of pSmad5 profiles are shown in **(D–F)**. Positions of expression boundaries for *sizzled* **(G)**, *foxi1* **(H)**, and *bambia* **(I)** are shown as vertical solid lines. Slope of pSmad5 at the boundary is indicated as a horizontal dotted line. Colored dots indicate positions where target genes are expressed. Standard deviations of expression boundaries are shaded. See Tables F–H in S3 Data for underlying data. A.U. is arbitrary units. **P < 0.01, ***P < 0.0001 in comparing pSmad5 levels and slopes using unpaired 2-tailed Student *t* tests. DV, dorsal–ventral; hpf, hours post fertilization; pSmad5, phosphorylated Smad5; WT, wild-type.

Next, we determined where the boundaries of the 3 BMP target genes are located along the pSmad5 gradient. We took sibling embryos of those stained for FISH and quantified pSmad5 immunostaining at an early gastrula (7 hpf) stage (Fig 3A). To identify the pSmad5 position that corresponds to each target gene expression boundary, we overlaid the DV position of the target gene boundaries onto the pSmad5 gradient profile of the sibling embryos. The pSmad5 gradient position at each expression boundary could indicate a distinct pSmad5 gradient slope or threshold that cells need to reach to induce differential expression of each gene. If gene expression is determined by a pSmad5 threshold level, then cells will not express a target gene until they reach the particular threshold level of pSmad5. Alternatively, if gene expression boundaries are positioned by distinct gradient slopes, then cells will activate target genes in response to a particular steep or shallow slope of the gradient independent of specific pSmad5 levels. It is also possible that 1 gene may respond to slope, while the others respond to distinct thresholds, for example. The same mechanism need not apply to all 3 target genes.

We found that expression of the 3 target genes correlates with significantly distinct pSmad5 gradient levels (Fig 3B). The expression boundary of *sizzled* corresponds to 60% of maximum pSmad5 intensity (Fig 3D). While the expression boundary of *foxi1* corresponds to 25% of maximum pSmad5 intensity (Fig 3E), that of *bambia* is very low at only 7% of pSmad5 maximum intensity (Fig 3F). The slope of the gradient changes across the DV axis in addition to the level of pSmad5. To determine if the expression boundaries correlate with distinct slopes of the pSmad5 gradient, we overlaid the boundaries of the target gene domains onto the slope of the pSmad5 gradient. The 3 target gene domains also correspond to 3 significantly distinct pSmad5 gradient slopes (Fig 3C). The expression boundary of *sizzled* corresponds to a gradient slope of 1.4 (A.U./degree), *foxi1* corresponds to a slope of 0.76 (A.U./degree), and *bambia* to a slope of 0.35 (A.U./degree) (Fig 3G–3I). To determine if cells along the AV axis show a differential responsiveness to BMP signaling, we analyzed the expression profiles of the 3 target genes over the top of the AV axis of the embryo (S8A Fig). The 3 targets are also expressed in significantly distinct profiles across the AV axis (S8B–S8D Fig), which correlates with significantly distinct pSmad5 levels (S8E–S8H Fig). The expression domain of *sizzled* corresponds to a distinct gradient slope, while the slopes at the *foxi1* and *bambia* boundaries are not significantly distinct over the AV axis (S8I–S8L Fig).

### Test of gradient slope to position gene expression boundaries

**Fig 4:**
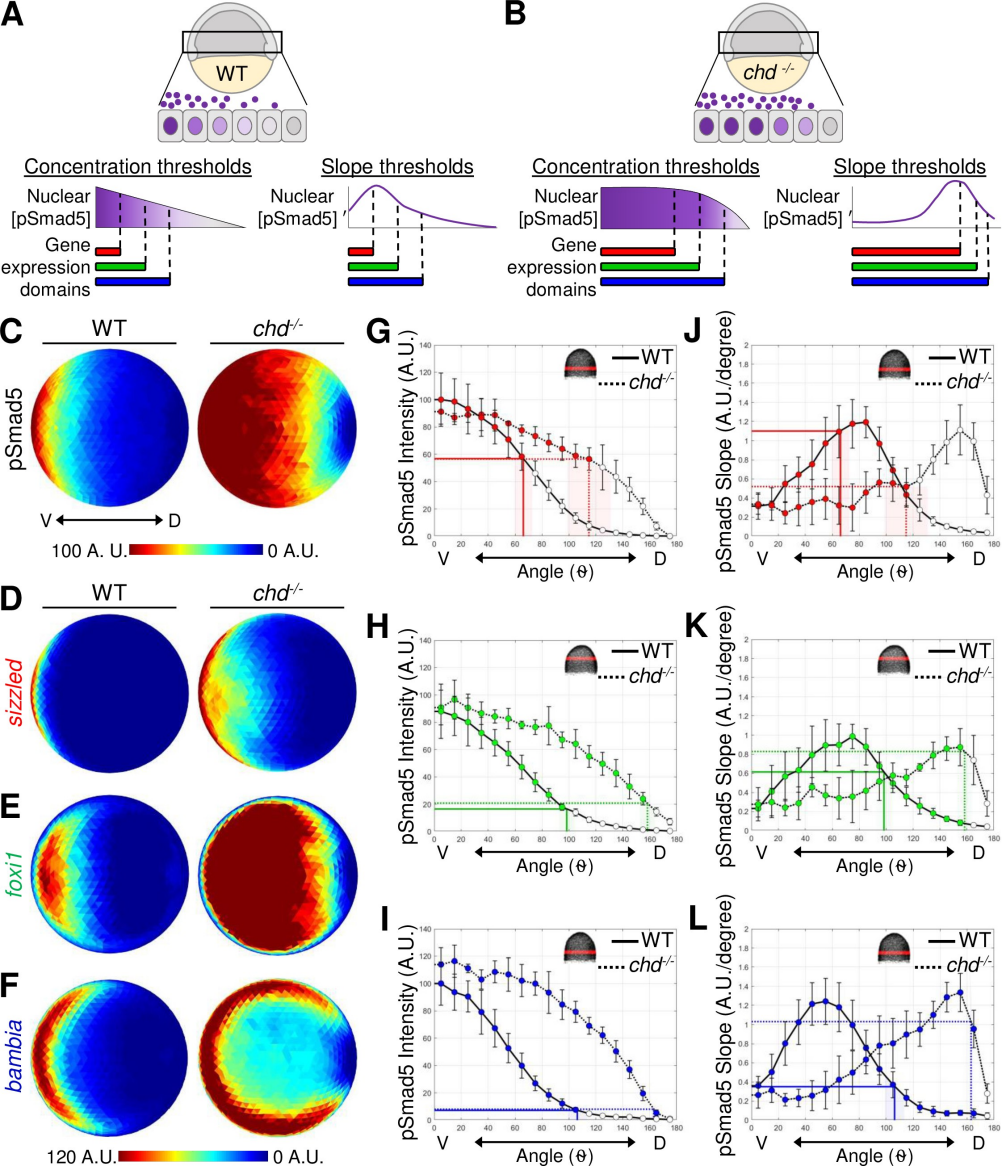
pSmad5 gradient thresholds versus slopes in positioning target gene expression in the *chordin* mutant gradient. **(A**) Model of 3 target gene expression domains in WT gastrula embryos positioned by either distinct pSmad5 levels or distinct pSmad5 gradient slopes. **(B)** Model of predicted target gene expression boundaries in *chordin* mutant gastrula embryos corresponding to distinct pSmad5 levels or gradient slopes if cells are interpreting pSmad5 concentration or shape, respectively. **(C)** Animal view of average pSmad5 intensities of early gastrula (7 hpf) WT (*n* = 6) and *chordin* mutants (*n* = 5). **(D–F)** Animal view of average FISH intensities of WT and *chordin* mutants for *sizzled* **(D)** (WT *n* = 8, *chd-/- n* = 8); *foxi1* **(E)** (WT *n* = 6, *chd-/- n* = 7); and *bambia* **(F)** (WT *n* = 9, *chd-/- n* = 4). **(G–I)** pSmad5 profiles of WT (black solid line) and *chordin* mutants (black dotted line). Location of 40-μm band of cells that was averaged is indicated on embryo in top right corner. Expression boundaries of *sizzled* **(G)**, *foxi1* **(H)**, and *bambia* **(I)** in WT (solid colored line) and *chordin* mutants (dotted colored line). Standard deviations of expression boundaries are shaded. See Tables A–C in S4 Data for underlying data. **(J–L)** Slope of pSmad5 profiles shown in **(G–I)** of WT (black solid line) and *chordin* mutants (black dotted line). Expression boundaries of *sizzled* **(J)**, *foxi1* **(K)**, and *bambia* **(L)** in WT (solid colored line) and *chordin* mutants (dotted colored line). Standard deviations of expression boundaries are shaded. See Tables D–F in S4 Data for underlying data. A.U. is arbitrary units. FISH, fluorescent in situ hybridization; hpf, hours post fertilization; pSmad5, phosphorylated Smad5; WT, wild-type.

The boundaries of target gene expression across the DV axis correspond to both distinct pSmad5 concentrations and pSmad5 gradient slopes. To directly test the ability of either pSmad5 concentration thresholds or gradient slope to position gene expression in the gastrula embryo, we utilized mutants that have a modified pSmad5 gradient shape and measured corresponding shifts in target gene expression (Fig 4A and 4B). If cells across the DV axis respond to distinct levels of pSmad5 to activate target gene expression, the boundaries of target gene expression will correlate with the same pSmad5 levels even if the gradient shape is altered (Fig 4B). Alternatively, if cells respond to the shape of the gradient, the target gene boundaries will correlate with the same gradient slope regardless of pSmad5 level (Fig 4B).

To investigate the spatial shifts in target gene expression when the pSmad5 gradient shape is altered, we used *chordin* mutant early gastrula embryos. Chordin is a BMP ligand antagonist that acts as a dorsal sink for BMP, thus shaping BMP signaling activity during gastrulation [23,35,63]. In *chordin* mutant embryos, the shape of the BMP signaling gradient is altered where the highest levels of BMP signaling activity expand laterally, and the slope of the gradient is shallower in the lateral regions (Fig 4C and 4G–4L, S9A Fig). While maximum pSmad5 levels are similar in wild-type and *chordin* mutant embryos during gradient formation, from 4.7 to 6.3 hpf [23], a broader region of cells express the highest pSmad5 levels in *chordin* mutants (Fig 4C and 4G–4I).

Embryos from crosses between *chordin-/-* and +/- fish were immunostained at an early gastrula stage (7 hpf) for pSmad5, while siblings were assayed by FISH for the 3 target genes (S9C–S9E Fig). The pSmad5 gradient and FISH domains were quantitated blindly, followed by genotyping for the *chordin* mutation. The gradient of pSmad5 is expanded laterally in *chordin* mutants compared to the wild-type siblings (Fig 4C), as previously shown [23]. The expression domains of the 3 target genes were also significantly expanded laterally in *chordin* mutants (Fig 4D–4F, S9B Fig).

To test whether a similar pSmad5 level delineated the boundary of the expression domains, possibly acting as a concentration threshold to provide positional information to cells, we determined the position of the target gene expression boundary on the pSmad5 gradients of wild-type and *chordin* mutant siblings. We found that a similar pSmad5 level corresponds to the boundary of *sizzled*, *foxi1*, and *bambia* expression in both wild-type and *chordin* mutants (Fig 4G–4I, S9F Fig). The *sizzled* boundary corresponds to 56.8% and 56.5% of the maximum pSmad5 intensity in wild-type and *chordin* mutants, respectively. The *foxi1* boundary corresponds to 16.5% and 20.8% in wild-type and *chordin* mutants. The *bambia* boundary corresponds to 7.2% and 8.0% in wild-type and *chordin* mutants. The similar levels of pSmad5 delineating the expression boundaries of these 3 target genes in wild-type and *chordin* mutants provides strong support for a concentration threshold model but does not eliminate the other models.

We also determined the pSmad5 gradient slope at the boundaries of the 3 target genes in both wild-type and *chordin* mutants. We did not find a consistent pSmad5 gradient slope at the expression boundaries for *sizzled* and *bambia* (Fig 4J and 4L, S9G Fig). The *sizzled* boundary corresponds to gradient slopes of 1.1 and 0.52 (A.U./degree) in wild-type and *chordin* mutants, respectively. The *bambia* boundary corresponds to 0.35 and 1.0 slopes (A.U./degree) in wild-type and *chordin* mutants. The boundary of *foxi1* expression corresponds to 0.61 and 0.83 slopes (A.U./degree) in wild-type and *chordin* mutants which is not significantly distinct (Fig 4K, S9G Fig). However, multiple positions across the DV axis have similar pSmad5 gradient slopes, so gradient shape alone would be unable to provide specific positional information. In contrast, the concentration threshold model does provide unique positional information across the DV axis.

### Test of signal duration to position gene expression boundaries

**Fig 5:**
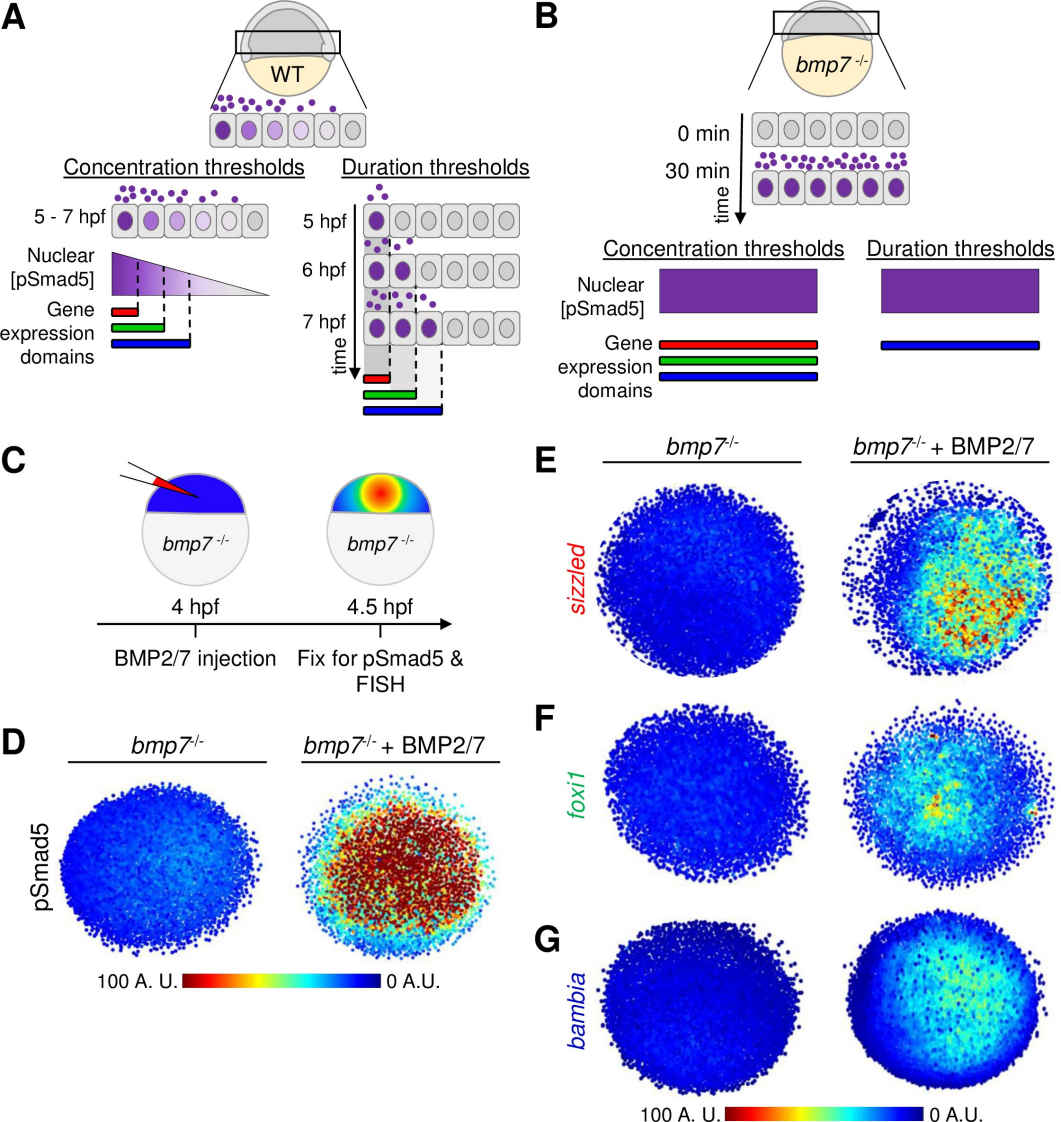
Thirty-minute duration of BMP2/7 sufficient for *sizzled*, *foxi1*, and *bambia* target gene expression. **(A)** Model of target gene expression regulated by distinct pSmad5 levels or distinct durations of BMP signaling. **(B)** Model of target gene activation after a 30-minute pulse of BMP ligand exposure. If target genes are activated by different pSmad5 levels, then all 3 target genes will be expressed following exposure to high levels of BMP signaling. If differences in signal duration activate BMP target gene expression, then a gene that requires a short signal duration will be expressed, but genes requiring longer signal durations will not be expressed. **(C)** Experimental schematic of a *bmp7* mutant embryo injected with 5 pg of BMP2/7 protein that is fixed 30 minutes postinjection for pSmad5 immunostaining or FISH. **(D)** Representative immunostaining of pSmad5 intensities of an uninjected *bmp7* mutant (*n* = 8) and a *bmp7* mutant injected with 5 pg of BMP2/7 protein (*n* = 9). Animal pole is facing up. **(E–G)** Representative FISH in *bmp7* mutants uninjected or injected with 5 pg of BMP2/7 protein for *sizzled* **(E)** (*n* = 11 uninjected, *n* = 11 injected), *foxi1* **(F)** (*n* = 10, *n* = 11), and *bambia* **(G)** (*n* = 10, *n* = 11). Animal pole is facing up. A.U. is arbitrary units. BMP, Bone Morphogenetic Protein; FISH, fluorescent in situ hybridization; hpf, hours post fertilization; pSmad5, phosphorylated Smad5; WT, wild-type.

The gradient of BMP signaling forms from mid-blastula to early gastrula stages [22,23,36]. Embryonic cells are exposed to the BMP2/7 ligand for over 4 hours during this time period. It is not known if the activation of target genes requires differential duration to BMP signaling activity or if cells activate target gene expression once the pSmad5 threshold level is reached (Fig 5A). In a signal duration model that determines ventral target gene expression boundaries, the most ventrally restricted genes would require the longest signal duration (e.g., *sizzled*), whereas the most broadly expressed ventrolateral target genes would require the shortest signal duration (e.g., *bambia*) (Fig 5A). To address the role of signal duration to pattern ventral cell fates, we tested the requirement of BMP ligand exposure to activate target gene expression. If cells respond to different durations of signal, then genes that require longer signal exposure will not be expressed after a pulse of BMP signaling. If cells respond to concentration thresholds, then a pulse of a high BMP2/7 concentration will activate all 3 target genes (Fig 5B).

To test the role of signal duration on the immediate response of gene expression, we injected BMP2/7 protein into *bmp7* mutant embryos at 4 hpf and fixed embryos 30 minutes after injection for FISH and pSmad5 immunostaining (Fig 5C). We detected robust pSmad5 activation after 30 minutes of BMP2/7 ligand exposure (Fig 5D). Strikingly, expression of *sizzled* (Fig 5E), *foxi1* (Fig 5F), and *bambia* (Fig 5G) was also observed 30 minutes postinjection. This suggests that spatially distinct target genes can be rapidly activated following exposure to BMP ligand. Specifically, the activation of these target genes does not require the full 4 hours of BMP signaling that cells are exposed to endogenously.

While even shorter signal durations would be unlikely to be physiologically relevant, we nevertheless investigated gene expression responses at shorter durations of 10, 20, and 30 minutes in this assay. We found that *sizzled* is expressed within 10 minutes of activating BMP signaling in *bmp7* mutant embryos (S10A and S10B Fig). The expression of *foxi1* and *bambia* was first observed 20 and 30 minutes after BMP2/7 injection, respectively (S10C and S10D Fig). While *bambia* with the broadest expression domain would be expected to require the shortest signal duration, we found that it was expressed latest among the 3 genes at 30 minutes in response to BMP signaling (S10 Fig).

To determine if genes in the same domain share the same transcriptional kinetics, we measured the expression of another broadly expressed target gene, *ved* (S11A Fig). The expression profiles of *ved* closely resemble *bambia* in both wild-type and *chordin* mutant embryos indicating that *ved* is activated by the same low pSmad5 threshold level (S11B Fig). However, unlike *bambia*, *ved* is rapidly activated in response to BMP signaling (S10D and S11C Figs). While differences in transcriptional kinetics have been suggested to underlie target genes activated by Nodal in zebrafish patterning [64], differences in the transcriptional kinetics of these BMP target genes do not correlate with domain size.

The mechanism of duration-dependent signaling can also include a genetic regulatory network that creates spatially distinct domains of expression [65]. To determine the role of secondary transcriptional regulation in defining the expression domains, we treated *bmp7* mutant embryos with CHX at 4 hpf before injecting BMP2/7 protein (S12A Fig). Again, all 3 target genes were rapidly activated with the CHX treatment after 30 minutes of BMP ligand exposure (S12B–S12D Fig). Thus, BMP target genes can be expressed in distinct domains of the embryo independent of distinct durations of BMP signaling or feedback through genetic regulatory networks. Therefore, we conclude that different durations of BMP signaling activity are not directly positioning the expression of these target genes within the embryo.

### Test of signal concentration to activate different target genes

**Fig 6:**
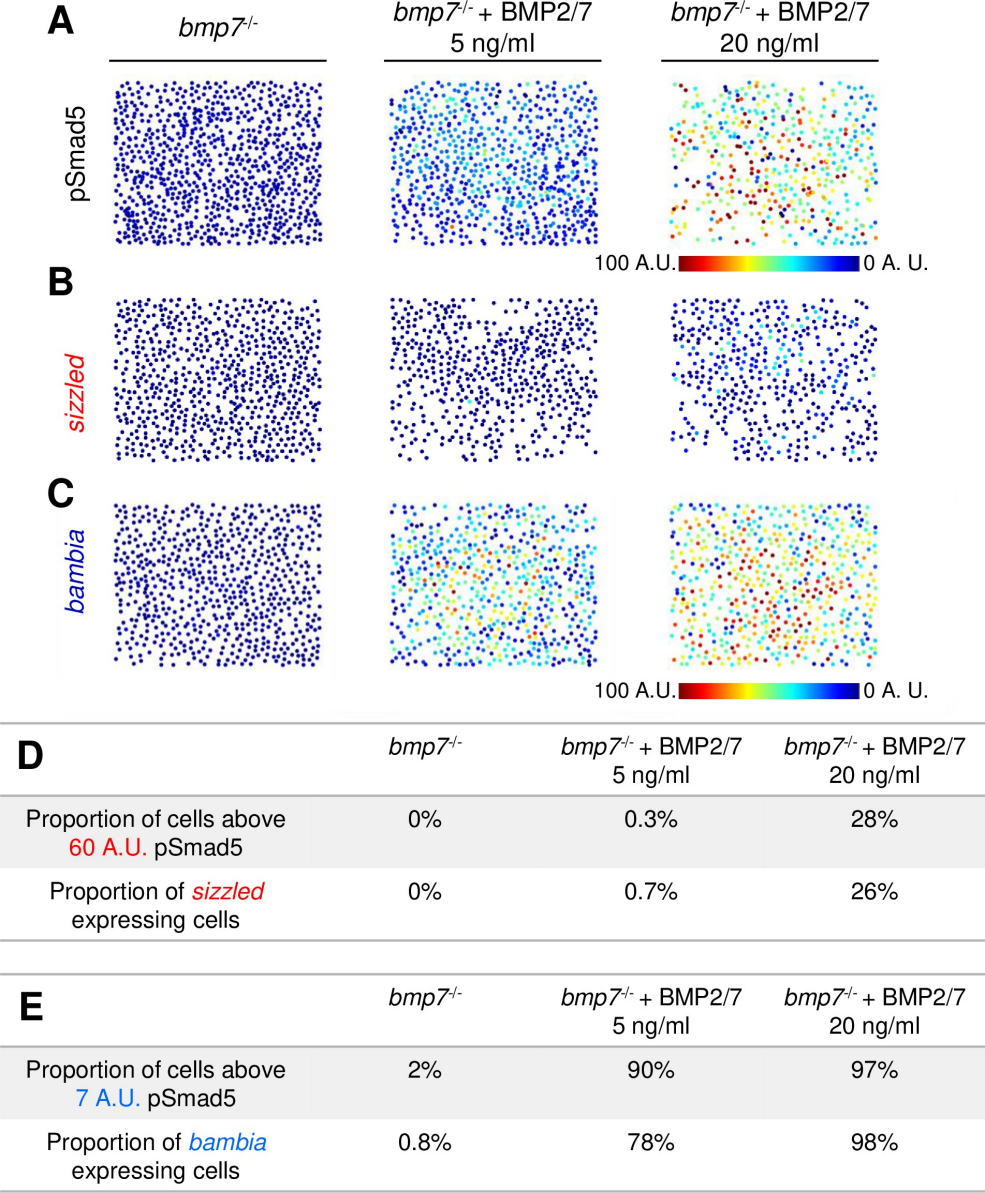
Target genes *sizzled* and *bambia* respond to BMP in a concentration-dependent manner. **(A)** Representative pSmad5 immunostaining intensities of disassociated cells from *bmp7* mutants and *bmp7* mutants treated with 5 or 20 ng/ml BMP2/7 protein. **(B, C)** Representative FISH for *sizzled* **(B)** and *bambia* **(C)** in *bmp7* mutants and *bmp7* mutants treated with 5 or 20 ng/ml BMP2/7 protein. **(D)** Table displaying the proportion of cells above the predicted pSmad5 threshold for *sizzled* (60 A.U.) in each condition, and the proportion of cells expressing *sizzled* in each condition. Cells with greater than 10% of maximum signal intensity are considered to be expressing *sizzled*. **(E)** Table displaying the proportion of cells above the predicted pSmad5 threshold for *bambia* (7 A.U.) in each condition, and the proportion of cells expressing *bambia* in each condition. Cells with greater than 10% of maximum signal intensity are considered to be expressing *bambia*. A.U. is arbitrary units. BMP, Bone Morphogenetic Protein; FISH, fluorescent in situ hybridization; pSmad5, phosphorylated Smad5.

We have shown that the 3 target genes are expressed rapidly upon exposure to high levels of BMP (Fig 5). If the target genes are responding to concentration thresholds alone, then exposing cells to different levels of BMP should activate target genes expressed in distinct domains regardless of signal duration or gradient shape. To expose cells to more stable and uniform levels of BMP, we manually disassociated cells from *bmp7* mutant animal caps at 4 hpf and incubated the disassociated cells with 20 ng/ml or 5 ng/ml BMP2/7 protein for 2 hours. Incubation of 20 ng/ml and 5 ng/ml BMP2/7 recapitulated endogenous high and low levels of BMP signaling, respectively, found in wild-type embryos (Fig 6A, S13 Fig). Cells from *bmp7* mutants expressed *sizzled* when incubated with the high level of BMP2/7 protein but not the lower level (Fig 6B, S14A Fig), as predicted in the concentration threshold model. Also consistent with the concentration threshold model, *bambia* is expressed in response to both high and low levels of BMP2/7 protein (Fig 6C, S14B Fig). Furthermore, these results show that a 2-hour duration of a low BMP signaling level cannot induce the expression of a high-threshold gene that responds within 10 minutes in the embryo (S10B Fig). Thus, the activation of a high-threshold target gene does not display a duration-based response to BMP signaling.

We further quantitated in this assay system the number of cells above each predicted pSmad5 threshold for *sizzled* and *bambia* expression and the number of cells expressing each gene (Figs 3D–3F and 4G–4I). The predicted pSmad5 threshold for *sizzled* is 60 A.U., and the predicted pSmad5 threshold is 7 A.U. for *bambia*. There is a similar proportion of cells with pSmad5 levels above the predicted *sizzled* threshold (60 A.U.) and cells expressing *sizzled* in *bmp7* mutants treated with 5 ng/ml and 20 ng/ml BMP2/7 (Fig 6D). Also, similar proportions of cells are above the predicted pSmad5 threshold level for *bambia* (7 A.U.) and are expressing *bambia* in the *bmp7* mutants treated with 5 ng/ml and 20 ng/ml BMP2/7 (Fig 6E). Together, our data support a model where distinct concentration thresholds of BMP signaling activate spatially distinct target genes in DV axial patterning during gastrulation.

## Discussion

### BMP morphogen interpretation: Multiple mechanisms for 1 signaling pathway

Multiple mechanisms have been reported for how a gradient of BMP signaling activity can provide positional information to pattern a tissue. In fact, there is conflicting evidence for how cells perceive gradients of BMP signaling depending on the context. In *Drosophila*, Dpp (the BMP homolog) is required for patterning and proliferation of the wing imaginal disc [66,67]. Cells in the wing disc have been suggested to sense the shape of the Dpp gradient [14] and the relative temporal change of Dpp signaling [68]. There is similar conflicting evidence for BMP patterning the dorsal neural tube. Both signal duration [10] and ligand identity [11] have been proposed to pattern neuronal identities. The mechanisms responsible for cellular interpretation of the BMP signaling gradient could vary in different contexts or by individual target genes. Understanding the mechanism of BMP morphogen interpretation in DV patterning will allow us to compare mechanisms across species and systems.

Here, we provide evidence that the BMP morphogen gradient acting to pattern DV axial tissues is interpreted in a concentration-dependent manner to activate 3 target genes underlying ventral cell fate specification. We precisely measured endogenous pSmad5 levels and gene expression activation within the embryo at a single-cell resolution. We found that the genes directly patterned by the BMP signaling gradient are expressed in at least 3 distinct domains of the embryo, which correspond to at least 3 different pSmad5 gradient levels that are required to activate representative target genes from each domain. Further, these pSmad5 levels act as thresholds that cells must reach to activate target gene expression and position gene expression boundaries in the embryo, regardless of gradient shape or exposure time. Together, our data support a model whereby the BMP signaling gradient is interpreted as a concentration-dependent morphogen providing positional information to pattern gene expression along the embryonic DV axis.

### Multiple expression domains directly patterned by the BMP gradient

The genes directly patterned by the BMP signaling gradient remained unknown prior to this work. Identification of the genes reading out the BMP gradient during DV patterning is not only critical to study the mechanism of morphogen patterning we report here, but also valuable for investigations of the ventral cell types specified by this morphogen gradient. Known markers of ventrally derived tissues were found to be directly regulated by BMP signaling such as *tp63* specifying epidermis [55,56], *foxi1* specifying otic placode [37,42], and *tfap2c* specifying neural crest precursors [57], as well as genes with unknown roles in cell fate specification. We identified many genes encoding components of BMP and other signaling pathways. Interestingly, BMP signaling directly activates the canonical Wnt receptors *fzd4/5*, the Nodal signaling cofactor *foxh1*, and the Retinoic Acid binding protein *crabp2b*. This pathway specific feedback may be critical for robust gradient formation and integration of multiple signaling pathways during embryonic patterning. While our analysis identified the initial gene expression readout of the BMP gradient, further work is needed to resolve the genetic regulatory network committing progenitors to specific ventral cell fates.

To determine the number of positional values established by the BMP signaling gradient, we reanalyzed a scRNA-seq dataset [58] using the Seurat analysis algorithm [59] to infer the DV expression domains of the 57 direct targets identified. Based on these results, the target genes were sorted into 3 DV clusters and validated by analysis of a representative of each cluster as expressed in 3 distinct embryonic domains. Although we identified 3 distinct domains, the remaining target genes could further partition into additional domains across the DV axis. These broad overlapping domains will undergo further refinement through regulatory feedback and interaction with other signaling pathways later in development to specify cell fates. For example, the BMP target gene and preplacodal ectodermal marker *dlx3b* is initially broadly expressed in cluster 3 (S4 Fig). By late gastrulation (9 hpf), expression of *dlx3b* is restricted by factors also induced by BMP signaling, *tfap2a/c*, *foxi1*, and *gata3* to form bilateral stripes that separate specification of epidermis and preplacodal ectoderm [37]. Future studies will have to address how other broadly expressed domains are refined into sharp, spatially discrete domains.

### Concentration, not gradient shape or duration, positions expression boundaries

Our mutant analysis demonstrates that cells do not interpret the pSmad5 gradient slope to activate 3 spatially distinct target genes. The slope of the pSmad5 gradient undergoes a 2-fold decrease in *chordin* mutants compared to wild-type embryos in the ventral–lateral region (25 to 75 degrees) and a 2-fold increase in the dorsal–lateral region (125 to 155 degrees) (Fig 4J–4L), but the boundaries of target gene expression remain strongly correlated with specific pSmad5 levels (Fig 4G–4I). Previous analysis of the BMP signaling gradient over time has revealed that the gradient is highly dynamic from mid-blastula to mid-gastrula stages [22,23]. Nuclear pSmad5 levels rapidly increase at the most ventral positions (0 to 25 degrees) from 4.7 to 6.7 hpf. However, the pSmad5 gradient region determined to pattern target gene expression (70 to 110 degrees) is remarkably stable during this time [23]. Our lab previously found no significant difference in pSmad5 levels in this region during this time period, although the gradient slope undergoes a 2-fold increase [23]. Stable pSmad5 levels over time may allow cells to continually read out the gradient to reduce cell-to-cell variability, and steepening of the slope could allow for sharper gene expression boundaries to form.

We also showed that cells do not require integration of BMP signaling over a prolonged duration for initial patterning of the DV axis. Further genetic regulatory networks may act over time to refine the gene expression domains. We found that cells rapidly activate multiple, distinctly expressed target genes upon a 30-minute exposure to the BMP ligand. This is consistent with previous studies from our lab showing that BMP signaling prior to gastrulation is not required for DV patterning [22]. Specifically, reinitiating BMP signaling activity at 6 hpf in a BMP-deficient embryo rescues DV patterning. However, reinitiating BMP signaling activity at 6.5 hpf in a BMP-deficient embryo failed to rescue. Therefore, while cells do not require integration of BMP signaling before gastrulation, there is a critical window of time in early gastrulation during which cells are responding to BMP signaling. Furthermore, all cells across the DV axis are exposed to BMP signaling for the same length of time, while the gradient forms [22,23]. During mid-blastula stages (before 4 hpf), BMP signaling is activated at low levels everywhere, before signaling is cleared from the dorsal side and the gradient steepens [22]. Together, these data show that cells do not require differences in BMP signal duration to activate spatially distinct target genes.

While BMP signal duration or gradient slope has been shown to pattern tissues in other contexts, we find that distinct pSmad5 threshold levels pattern the embryonic DV axis. It will be important to investigate the molecular mechanism by which these thresholds activate target genes to compare BMP interpretation across contexts. The molecular mechanisms that establish different pSmad5 thresholds will be particularly interesting because BMP-responsive Smad transcription factors bind DNA with weak affinity [69,70]. Therefore, the classic model where differential affinity of transcription factors to regulatory elements produces spatially distinct target gene expression patterns alone cannot underlie BMP interpretation. To increase affinity and selectivity for DNA, Smad proteins bind to other high-affinity DNA-binding transcription factors [71]. Differential DNA-binding of Smad to target gene regulatory elements may be mediated by interactions with these cofactors. In *Drosophila*, the cofactor Zelda is required for BMP target genes to be expressed in the correct domain [72] and may represent such a Smad cofactor. However, a Zelda ortholog is not present in vertebrates, so other cofactors may play this role. Future studies on the association of Smad5 with cofactors or chromatin modifiers will be essential to further uncover the mechanism establishing concentration-dependent morphogen interpretation.

## Materials and methods

### Zebrafish

Adult zebrafish (*Danio rerio*) were kept at 28°C in a 13-hour light/11-hour dark cycle, and procedures were approved by the University of Pennsylvania Institutional Animal Care and Use Committee (IACUC). All zebrafish husbandry were performed in accordance with institutional, national ethical, and animal welfare guidelines. The embryos used for experiments were between 0 and 8 hpf, with some phenotypes tracked 1 to 2 days post fertilization. Embryos were collected and raised at 28°C in E3 solution. In this study, sex/gender is not relevant since zebrafish sex determination takes place after 25 days post fertilization [73]. The zebrafish lines used were WT (TU) (RRID: ZIRC_ ZL57), *chordin*^*tt250*^ (RRID: ZDB-ALT-980413-523, ZIRC_ZL61), and *bmp7a*^*sb1aub*^ (RRID: ZFIN_ZDB-ALT-050216-2, ZIRC_ZL1390). Adult *chordin*^*tt250*^ homozygous fish were generated by injecting *chordin* mRNA into 1-cell stage *chordin*-/- embryos to rescue the embryonic ventralization and then raised to adulthood. Adult *bmp7a*^*sb1aub*^ homozygous fish were used to produce all *bmp7a*^*sb1aub*^ homozygous embryos. Adult *bmp7a*^*sb1aub*^ homozygotes were generated by injecting *bmp7a* mRNA into 1-cell stage *bmp7a*-/- embryos to rescue the embryonic dorsalization and then raised to adulthood.

### Genotyping

Adult and embryonic genomic DNA was obtained using HotShot DNA isolation. Genotyping of adults and embryos for the *chordin* mutation was performed using KASPar genotyping [74]. Primers were designed and generated by LGC BiosearchTechnologies (Petaluma, California, United States of America) to the following sequences flanking the [WT/ *chordin*^*tt250*^] nucleotide: GTTTGGTGTGATGCACTGCGTTATGTGTCATTGTGAGCCG[G/A]TGAGTTGTGCACAGTTCAGTTTGAAATCCATATTGAATCT.

### pSmad5 immunostaining, imaging, and quantification

pSmad5 immunostaining, imaging, and quantification were performed as previously described [23,75]. Embryos were fixed in 4% paraformaldehyde at 4°C, blocked in NCS-PBST and probed overnight with a 1:100 dilution of anti-phosphoSmad1/5/9 antibody (Cell Signaling Technology, Cat# 13820, RRID:AB_2493181, Danvers, Massachusetts, USA), followed by a 1:500 dilution of goat anti-rabbit Alexa Fluor 647 (Molecular Probes, Cat# A-21244, RRID:AB_141663, Eugene, Oregon, USA) and a 1:1,000 dilution of Sytox Green (Thermo Fisher Scientific, Cat# S7020, Waltham, Massachusetts, USA). Embryos were gradually dehydrated into methanol, then cleared and mounted in BABB, a 1:2 ratio of benzyl alcohol (Sigma-Aldrich, B-1042, St. Louis, Missouri, USA) and benzyl benzoate (Sigma-Aldrich, B-6630, St. Louis, Missouri, USA). Mounted embryos were imaged on a Zeiss LSM880 confocal microscope with an LD LCI Plan-Achromat 25X/0.8 lmm Corr DIC M27 multi-immersion lens in the oil-immersion setting (Zeiss, Oberkochen, Germany). The same single bead from a calibration slide (Thermo Fisher Scientific, Cat#F369009, well A1, Waltham, Massachusetts, USA) was imaged between slides to account for any fluctuations in laser power.

Images were analyzed with a custom MATLAB algorithm to identify individual nuclei center points and extract pSmad5 intensities from within each nucleus [23,75], which were normalized based on the standard calibration bead intensity. Resulting embryos were aligned across the DV axis and conformed using Coherent Point Drift. Population means were generated after genotyping for wild-type and heterozygous sibling controls, since all imaging and analysis was performed blinded. Mean profiles were generated by averaging pSmad5 intensities of cells in a 40-μm band. Three-dimensional (3D) embryo-wide displays of mean pSmad5 were generated by projecting all nuclei on a sphere divided into 4,800 equilateral triangles and nuclei within each triangle averaged together. The pSmad5 gradient slopes were obtained by fitting a lowess fit to the average 3D data’s spherical coordinates phi and theta using the “fit” function in MATLAB.

### Fluorescent in situ hybridization, imaging, and quantification

Embryos were fixed in 4% paraformaldehyde at 4°C and gradually dehydrated in methanol. Whole-mount in situ hybridizations were performed using DIG-labeled anti-sense RNA probes (made with labeling kit: Roche, 11175033910, Basel, Switzerland) to *sizzled* [76], *foxi1* [37], and *bambia*. Probes were visualized with anti-DIG-Horseradish Peroxidase (Roche, 11207733910, Basel, Switzerland) developed with TSA Plus Cyanine 3 kits using a 1:50 dilution (Perkin Elmer, NEL744001KT, Waltham, Massachusetts, USA), and nuclei were stained with 1:1,000 dilution of Sytox Green. Embryos were cleared, mounted, and imaged as described for immunofluorescence. Images were analyzed using the same MATLAB algorithm, except fluorescent intensity was extracted from a 25-pixel sphere from the center point of each nucleus to include the cytoplasmic staining.

### BMP2/7 protein injection

For CHX assays, embryos were dechorionated and treated with 10 μg/ml of CHX (Sigma-Aldrich, #C4859, St. Louis, Missouri, USA) at 4 hpf for 30 minutes. For both the CHX assay and time course, the embryos were injected with a 3-nl solution containing KCL (0.1 M), bovine serum albumin (BSA; 0.1%), rhodamine-dextran (0.5%), and either 120 or 60 nM of hBMP2-hBMP7 heterodimer (R&D Systems, 2339-BM, Minneapolis, Minnesota, USA) into the extracellular space. Embryos were allowed to develop for the time indicated, then fixed in 4% paraformaldehyde and processed for RNA extraction, whole-mount pSmad5 immunostaining, or FISH.

### Cell disassociation cultures

At 4 hpf, *bmp7* mutant embryos were dechorionated and placed into 1X Modified Barth’s Saline (MBS) as previously described [77]. One hundred animal caps were removed with forceps by cutting the blastoderm at approximately 50% of its height, and the collected cells were diluted to a final concentration of 5 × 10^5^ in 1X MBS containing Gentamicin (50 ug/ml; Gibco, Carlsbad, California, USA). The cells were disassociated by quickly vortexing, and 5 or 20 ng/ml of hBMP2-hBMP7 heterodimer (R&D Systems 2339-BM) was added to the tube for 2 hours before fixing with 4% paraformaldehyde. To perform immunofluorescence and FISH, cells were transferred to glass slides by cytospin at 750 rpm for 5 minutes. The pSmad5 immunostaining was performed and analyzed as described above with the slides mounted in Fluoromount-G (Southern Biotechnology Associates, Birmingham, Alabama, USA) for imaging. For FISH, cells were fixed in 4% paraformaldehyde for 10 minutes and allowed to air-dry for 1 hour before performing the FISH and analyzed as described above with the slides mounted in Vectashield mounting medium (Vector Labs, Burlingame, California, USA) for imaging. Fluorescent signal was normalized to the median of the top and bottom 5% of cells in wild-type early gastrula embryos imaged on slides.

### RNA sequencing and analysis

Two replicates of 40 wild-type and *bmp7* mutants were collected at early gastrula (shield, 6 hpf) and mid-gastrula (70% epiboly, 8 hpf). Three replicates of 50 *bmp7* mutant embryos treated with CHX with or without injected BMP2/7 protein were collected 90 minutes after injection. Total RNA was extracted from dechorionated embryos with Trizol and purified with phenol-chloroform. Libraries were prepared with Illumina TruSeq stranded polyA-selection mRNA kit (Illumina, San Diego, California, USA) by the Next-Generation Sequencing Core at the University of Pennsylvania. Libraries were analyzed using the Agilent BioAnalyzer (Agilent, Santa Clara, California, USA) and Kapa Biosystems library quantitation kit (Roche, Basel, Switzerland) before sequencing on a HiSeq4000. Sequence reads were aligned to the zebrafish GRCz11 genome assembly with RNA-seq Unified Mapper (RUM) [78]. Differential expression was determined with EdgeR, and values were normalized to counts per million. GO analysis of BMP target genes was performed using the PANTHER classification system for the enrichment analysis (http://pantherdb.org/) and Fisher exact test with Bonferroni correction and *P* < 0.05 to determine terms that are statistically significant [79,80].

### Seurat analysis of single-cell RNA sequencing dataset

Previously published scRNA sequencing from 6,100 individual cells dissociated from 75% epiboly embryos were used [58]. Cells with less than 2,000 genes sequenced were excluded from the analysis. Genes sequenced in fewer than 3 cells were also excluded from the analysis. Locations of the 47 landmark genes used are shown in [59]. Expression of target genes were mapped into bins using the Seurat package version 1.2 [59]. Data were normalized in Seurat.

### Statistical analysis

Statistical tests were performed on GraphPad Prism software, and Student *t* tests (2 groups) or analysis of variance (3 groups) were performed. Error bars represent standard deviation. Figure legends indicate the number of *n* values for each analysis. Each experiment was performed at least 2 times.

### Ethics statement

All animal procedures and protocols were performed in accordance with the approved IACUC protocols (#803105 and #804214) of the University of Pennsylvania and with the recommendations in the Guide for the Care and Use of Laboratory Animals of the National Institutes of Health.

## Supporting information

S1 Fig Identification of genes dependent on and directly regulated by BMP signaling. Related to Fig 1. **(A)** Animal view of the maximum projection of pSmad5 immunofluorescence of a WT and *bmp7* mutant mid-gastrula stage (8 hpf) embryo. Nuclei are stained with Sytox Green. **(B)** Mean pSmad5 profiles of WT (*n* = 4) (solid line) and *bmp7* mutants (*n* = 3) (dotted line) across the DV axis. Location of the 40-μm band of cells (red) that was averaged is indicated on the embryo in top right corner. See Table D in S1 Data for underlying data. **(C)** Differential gene expression of WT and *bmp7* mutants at early gastrulation (6 hpf) using RNA-seq. Significantly up-regulated genes in WT compared to *bmp7* mutants shown in red, and significantly down-regulated genes are shown in blue. All other genes are shown in gray. A subset of known BMP-dependent genes is highlighted. See Table E in S1 Data for underlying data. **(D)** Animal view of in situ hybridization of *foxi1*, a known direct target of BMP signaling, in the conditions shown. **(E)** GO term analysis for molecular functions of the 57 direct target genes. See Table F in S1 Data for underlying data. A.U. is arbitrary units. BMP, Bone Morphogenetic Protein; CHX, cycloheximide; DV, dorsal–ventral; GO, Gene Ontology; hpf, hours post fertilization; pSmad5, phosphorylated Smad5; RNA-seq, RNA sequencing; WT, wild-type.
(TIF)

S2 Fig Seurat predicted expression domains of BMP directly up-regulated target genes in cluster 1. Related to Fig 2. Heat map of gene expression patterns from Seurat analysis for cluster 1 target genes directly up-regulated by BMP signaling and sequenced in scRNA-seq dataset of mid-gastrula (8 hpf) embryos. Cluster 1 target genes are expressed within the first 3 or 4 bins. Predicted expression normalized across all bins. Below are Seurat predicted expression profiles across the DV axis. Each point is the sum of the expression intensity from all bins at 1 DV position. Genes are considered expressed in a bin with greater than 0.5 A.U. of predicted expression. The genes known to expressed dorsally or in the prechordal plate are indicated by asterisks. See Table E in S2 Data for underlying data. A.U. is arbitrary units. BMP, Bone Morphogenetic Protein; DV, dorsal–ventral; hpf, hours post fertilization; scRNA-seq, single-cell RNA sequencing.
(TIF)

S3 Fig Seurat predicted expression domains of BMP directly up-regulated target genes in cluster 2. Related to Fig 2. Heat map of gene expression patterns from Seurat analysis for cluster 2 target genes directly up-regulated by BMP signaling and sequenced in scRNA-seq dataset of mid-gastrula (8 hpf) embryos. Cluster 2 target genes are expressed within the first 5 bins. Predicted expression normalized across all bins. Below are Seurat predicted expression profiles across the DV axis. Each point is the sum of the expression intensity from all bins at 1 DV position. Genes are considered expressed in a bin with greater than 0.5 A.U. of predicted expression. The genes known to expressed dorsally or in the prechordal plate are indicated by asterisks. See Table F in S2 Data for underlying data. A.U. is arbitrary units. BMP, Bone Morphogenetic Protein; DV, dorsal–ventral; hpf, hours post fertilization; scRNA-seq, single-cell RNA sequencing.
(TIF)

S4 Fig Seurat predicted expression domains of BMP directly up-regulated target genes in cluster 3. Related to Fig 2. Heat map of gene expression patterns from Seurat analysis for cluster 3 target genes directly up-regulated by BMP signaling and sequenced in scRNA-seq dataset of mid-gastrula (8 hpf) embryos. Cluster 3 target genes are expressed within the first 6 or 7 bins. Predicted expression normalized across all bins. Below are Seurat predicted expression profiles across the DV axis. Each point is the sum of the expression intensity from all bins at 1 DV position. Genes are considered expressed in a bin with greater than 0.5 A.U. of predicted expression. The genes known to expressed dorsally or in the prechordal plate are indicated by asterisks. See Table G in S2 Data for underlying data. A.U. is arbitrary units. BMP, Bone Morphogenetic Protein; DV, dorsal–ventral; hpf, hours post fertilization; scRNA-seq, single-cell RNA sequencing.
(TIF)

S5 Fig Seurat predicted expression domains of BMP directly up-regulated target genes in cluster 4. Related to Fig 2. Heat map of gene expression patterns from Seurat analysis for cluster 4 target genes directly up-regulated by BMP signaling and sequenced in scRNA-seq dataset of mid-gastrula (8 hpf) embryos. Cluster 4 target genes have random or uniform expression across the DV axis. Predicted expression normalized across all bins. Below are Seurat predicted expression profiles across the DV axis. Each point is the sum of the expression intensity from all bins at 1 DV position. Genes are considered expressed in a bin with greater than 0.5 A.U. of predicted expression. See Table H in S2 Data for underlying data. A.U. is arbitrary units. BMP, Bone Morphogenetic Protein; DV, dorsal–ventral; hpf, hours post fertilization; scRNA-seq, single-cell RNA sequencing.
(TIF)

S6 Fig Seurat predicted expression domains of 11 BMP directly down-regulated target genes. Related to Fig 2. Heat map of gene expression patterns from Seurat analysis for 11 target genes directly down-regulated by BMP signaling that were sequenced in a scRNA-seq dataset of mid-gastrula (8 hpf) embryos. Predicted expression was normalized across all bins. Below are Seurat predicted expression profiles across the DV axis. Each point is the sum of the expression intensity from all bins at 1 DV position. Genes are considered expressed in a bin with greater than 0.5 A.U. of predicted expression. Genes with higher predicted expression in bins 5–8 than bins 1–4 are considered to be dorsally enriched. See Table I in S2 Data for underlying data. A.U. is arbitrary units. BMP, Bone Morphogenetic Protein; DV, dorsal–ventral; hpf, hours post fertilization; scRNA-seq, single-cell RNA sequencing.
(TIF)

S7 Fig Quantifying FISH of 3 target genes. Related to Fig 2. **(A)** Animal view of a maximum projection of FISH for *sizzled*, *foxi1*, and *bambia* of individual WT embryos at an early gastrula stage (7 hpf). Nuclei are stained with Sytox Green. **(B)** Schematic of cells of individual embryo partitioned into 128 equally spaced bins. Expression intensity within each bin is averaged. Both halves of the embryo are averaged together into 64 bins, and the expression intensity is normalized across all bins. Expression intensity is displayed as an 8 by 8 heat map. **(C–E)** Individual (colored) and averaged (black) expression profiles of *sizzled* (*n* = 5) **(C)**, *foxi1* (*n* = 5) **(D)**, and *bambia* (*n* = 9) **(E)** across the DV axis of WT embryos at 7 hpf. Location of the 40-μm band of cells that was averaged is indicated on the embryo in the top right corner. The boundary of the expression domain was measured in individual embryos at the position of 10% maximum expression intensity. A.U. is arbitrary units. See Tables J–L in S2 Data for underlying data. DV, dorsal–ventral; FISH, fluorescent in situ hybridization; hpf, hours post fertilization; WT-wild-type.
(TIF)

S8 Fig Expression profiles across the AV axis of the embryo. Related to Fig 3. **(A)** Schematic showing the location where a band of cells over the top of the embryo was averaged, beginning at the ventral margin (−100^o^) and ending at the dorsal margin (100^o^). **(B–D)** Individual (colored) and averaged (black) expression profiles of *sizzled* (*n* = 5) **(B)**, *foxi1* (*n* = 5) **(C)**, and *bambia* (*n* = 9) **(D)** across the AV axis of WT embryos at 7 hpf. Location of the 40-μm band of cells that was averaged is indicated in red on the embryo in the top right corner. The boundary of the expression domain was measured in individual embryos at the position of 10% maximum expression intensity. See Tables I–K in S3 Data for underlying data. **(E)** Measurement of pSmad5 intensity at the location of expression boundaries for *sizzled* (red), *foxi1* (green), and *bambia* (blue) across the AV axis of WT embryos at 7 hpf. See Table L in S3 Data for underlying data. **(F–H)** pSmad5 profiles across the AV axis. The intensity is averaged from a 40-μm band of cells around the AV axis at the location shown in red in the right corner embryo schematic of each panel. One WT clutch was used for **(F, G)** (*n* = 5), and another clutch was used for **(H)** (*n* = 6). Positions of expression boundaries for *sizzled* **(F)**, *foxi1* **(G)**, and *bambia* **(H)** are shown as vertical solid lines. Level of pSmad5 at the boundary is indicated as a horizontal dotted line. Colored dots indicate positions where target genes are expressed. See Tables M and N in S3 Data for underlying data. **(I)** Measurement of pSmad5 slope at the location of expression boundaries for *sizzled* (red), *foxi1* (green), and *bambia* (blue) across the AV axis of WT embryos at 7 hpf. See Table O in S3 Data for underlying data. **(J–L)** Slopes of pSmad5 profiles are shown in **(F–H)**. Positions of expression boundaries for *sizzled* **(J)**, *foxi1* **(K)**, and *bambia* **(L)** are shown as vertical solid lines. Slope of pSmad5 at the boundary is indicated as a horizontal dotted line. Colored dots indicate positions where target genes are expressed. See Tables P and Q in S3 Data for underlying data. A.U. is arbitrary units. *P < 0.05, **P < 0.01, ***P < 0.0001 in comparing pSmad5 levels and slopes using unpaired 2-tailed Student *t* tests. NS is not significant. AV, animal–vegetal; hpf, hours post fertilization; pSmad5, phosphorylated Smad5; WT, wild-type.
(TIF)

S9 Fig pSmad5 levels and target gene expression in *chordin* mutants. Related to Fig 4. **(A)** Animal view of the maximum projection of pSmad5 immunofluorescence in an individual WT and *chordin* mutant at an early gastrula stage (7 hpf). Merged image with Sytox Green staining nuclei. Because the *chordin* mutant displays high pSmad5 levels throughout the embryo, a lower confocal laser intensity gain was used in imaging the WT and *chordin* mutant embryos in this experiment compared to other pSmad5 imaging experiments. **(B)** Position of expression boundaries for *sizzled* (red), *foxi1* (green) and *bambia* (blue) in individual WT (filled) and *chordin* mutant (opened) embryos. See Table G in S4 Data for underlying data. **(C–E)** Average expression profiles of *sizzled* **(C)**, *foxi1* **(D)**, and *bambia* **(E)** across the DV axis of WT (solid line) and *chordin* mutant (dotted line) embryos. Location of the 40-μm band of cells that was averaged is indicated on the embryo in the top right corner. See Tables H–J in S4 Data for underlying data. **(F)** Measurement of pSmad5 intensity at the location of expression boundaries for *sizzled* (red), *foxi1* (green), and *bambia* (blue) across the DV axis of WT (filled) and *chordin* mutant (opened) embryos at 7 hpf. See Table K in S4 Data for underlying data. **(G)** Measurement of pSmad5 gradient slope at the location of expression boundaries for *sizzled* (red), *foxi1* (green), and *bambia* (blue) across the DV axis of WT (filled) and *chordin* mutant (opened) embryos at 7 hpf. See Table L in S4 Data for underlying data. *P < 0.05, ***P < 0.001 in comparing DV position, pSmad5 levels, and pSmad5 slopes using unpaired 2-tailed Student *t* tests. NS is not significant. A.U. is arbitrary units. DV, dorsal–ventral; hpf, hours post fertilization; pSmad5, phosphorylated Smad5; WT, wild-type.
(TIF)

S10 Fig Differential target gene induction at 10, 20, and 30 minutes. Related to Fig 5. **(A)** Representative immunostaining of pSmad5 intensities of an uninjected *bmp7* mutant (*n* = 10) and *bmp7* mutants injected with 5 pg of BMP2/7 protein and fixed after 10, 20, and 30 minutes after injection (*n* = 10, *n* = 10, *n* = 10). Animal pole is facing up. **(B–D)** Representative FISH for *sizzled* **(E)** (*n* = 10, *n* = 11, *n* = 10, *n* = 10), *foxi1* **(F)** (*n* = 5, *n* = 5, *n* = 5, *n* = 5), and *bambia* **(G)** (*n* = 5, *n* = 5, *n* = 5, *n* = 5) in uninjected *bmp7* mutants and *bmp7* mutants injected with 5 pg of BMP2/7 protein and fixed 10, 20, and 30 minutes after injection. Animal pole is facing up. A.U. is arbitrary units. BMP, Bone Morphogenetic Protein; FISH, fluorescent in situ hybridization; pSmad5, phosphorylated Smad5.
(TIF)

S11 Fig Broadly expressed target gene *ved* rapidly activated following BMP exposure. Related to Fig 5. **(A)** Animal views of average FISH signal of *ved* in WT embryos (*n* = 6) and *chordin* mutants (*n* = 5) at an early gastrula stage (7 hpf). **(B)** Average expression profiles of *ved* (black) and *bambia* (blue) in WT (solid line) and *chordin* mutants (dotted line). Location of 40-μm band of cells that was averaged is indicated on embryo in right corner. See Table A in S5 Data for underlying data. **(C)** Representative FISH for *ved* in *bmp7* mutants uninjected or injected with 5 pg of BMP2/7 protein and fixed 10, 20, and 30 minutes after injection (*n* = 5, *n* = 5, *n* = 5, *n* = 5). Animal pole is facing up. A.U. is arbitrary units. BMP, Bone Morphogenetic Protein; FISH, fluorescent in situ hybridization; hpf, hours post fertilization; WT, wild-type.
(TIF)

S12 Fig Rapid activation of target genes does not require transcriptional feedback. Related to Fig 5. **(A)** Representative immunostaining of pSmad5 intensities of an uninjected *bmp7* mutant treated with CHX (*n* = 15) and a *bmp7* mutant treated with CHX and then injected with 5 pg of BMP2/7 protein (*n* = 15). Animal pole is facing up. **(B–D)** Representative FISH in CHX-treated *bmp7* mutants that were either uninjected or injected with 5 pg of BMP2/7 protein for *sizzled* **(E)** (*n* = 10 uninjected, *n* = 11 injected), *foxi1* **(F)** (*n* = 10, *n* = 10), and *bambia* **(G)** (*n* = 10, *n* = 11). Animal pole is facing up. A.U. is arbitrary units. BMP, Bone Morphogenetic Protein; CHX, cycloheximide; FISH, fluorescent in situ hybridization; pSmad5, phosphorylated Smad5.
(TIF)

S13 Fig Activation of different pSmad5 levels in BMP-treated disassociated cells. Related to Fig 6. **(A)** Representative maximum projection of pSmad5 immunofluorescence in whole-mount WT embryo, disassociated cells from *bmp7* mutants and disassociated cells from *bmp7* mutants treated with 5 or 20 ng/ml BMP2/7 protein. Merged image with Sytox Green stained nuclei. **(B)** Quantification of nuclear pSmad5 intensities of individual cells in whole-mount WT embryos, disassociated cells from *bmp7* mutants and disassociated cells from *bmp7* mutants treated with 5 or 20 ng/ml BMP2/7 protein. See Table A in S6 Data for underlying data. A.U. is arbitrary units. BMP, Bone Morphogenetic Protein; pSmad5, phosphorylated Smad5; WT, wild-type.
(TIF)

S14 Fig Differential response of *sizzled* and *bambia* to BMP concentrations. Related to Fig 6. **(A)** Representative maximum projection of *sizzled* FISH in whole-mount WT embryos, disassociated cells from *bmp7* mutants and disassociated cells from *bmp7* mutants treated with 5 or 20 ng/ml BMP2/7 protein. Merged image with Sytox Green stained nuclei. **(B)** Representative maximum projection of *bambia* FISH in whole-mount WT embryos, disassociated cells from *bmp7* mutants and disassociated cells from *bmp7* mutants treated with 5 or 20 ng/ml BMP2/7 protein. Merged image with Sytox Green stained nuclei. BMP, Bone Morphogenetic Protein; FISH, fluorescent in situ hybridization; WT, wild-type.
(TIF)

S1 Table BMP up-regulated target genes. Related to Fig 1 and S1 Fig. List of names and RefSeq accession numbers of genes directly activated by BMP signaling during gastrulation, as well as the corresponding cluster number based on the Seurat predicted expression profile. Clusters 1, 2, and 3 are genes with predicted ventrally enriched expression. Cluster 4 contains the genes with predicted uniform profiles or significant dorsal expression. NS indicates genes that were not sequenced in the Farrell et al. (2018) scRNA-seq dataset. BMP, Bone Morphogenetic Protein; scRNA-seq, single-cell RNA sequencing.
(DOCX)

S2 Table BMP down-regulated target genes. Related to S6 Fig. List of names and RefSeq accession numbers of genes directly inhibited by BMP signaling during gastrulation, as well as the expression domain predicted by Seurat. NE indicates predicted expression is not enriched along the DV axis. NS indicates genes that were not sequenced in the Farrell et al. (2018) scRNA-seq dataset. BMP, Bone Morphogenetic Protein; DV, dorsal–ventral; scRNA-seq, single-cell RNA sequencing.
(DOCX)

S1 Data Data underlying Fig 1B, 1E and 1F and S1B, S1C, and S1E Fig. (XLSX)

S2 Data Data underlying Fig 2A, 2B, 2C and 2K and S2–S6 and S7C–S7E Figs. (XLSX)

S3 Data Data underlying Fig 3B–3I and S8B–S8L Fig. (XLSX)

S4 Data Data underlying Fig 4G–4L and S9B–S9G Fig. (XLSX)

S5 Data Data underlying S11B Fig. (XLSX)

S6 Data Data underlying S13B Fig. (XLSX)

## Abbreviations

3D: three-dimensional
AP: anterior–posterior
AV: animal–vegetal
BMP: Bone Morphogenetic Protein
BSA: bovine serum albumin
CHX: cycloheximide
DV: dorsal–ventral
FDR: false discovery rate
FGF: Fibroblast Growth Factor
FISH: fluorescent in situ hybridization
GO: Gene Ontology
hpf: hours post fertilization
IACUC: Institutional Animal Care and Use Committee
MBS: Modified Barth’s Saline
pSmad5: phosphorylated Smad5
RNA-seq: RNA sequencing
RUM: RNA-seq Unified Mapper
scRNA-seq: single-cell RNA sequencing
Shh: Sonic Hedgehog
