# Supplementary figures and images for: The BMP signaling gradient is interpreted through concentration thresholds in dorsal–ventral axial patterning
Source: PLoS Biol. 2021 Jan 22;19(1):e3001059. doi: 10.1371/journal.pbio.3001059 (PMC7857602; doi:10.1371/journal.pbio.3001059)

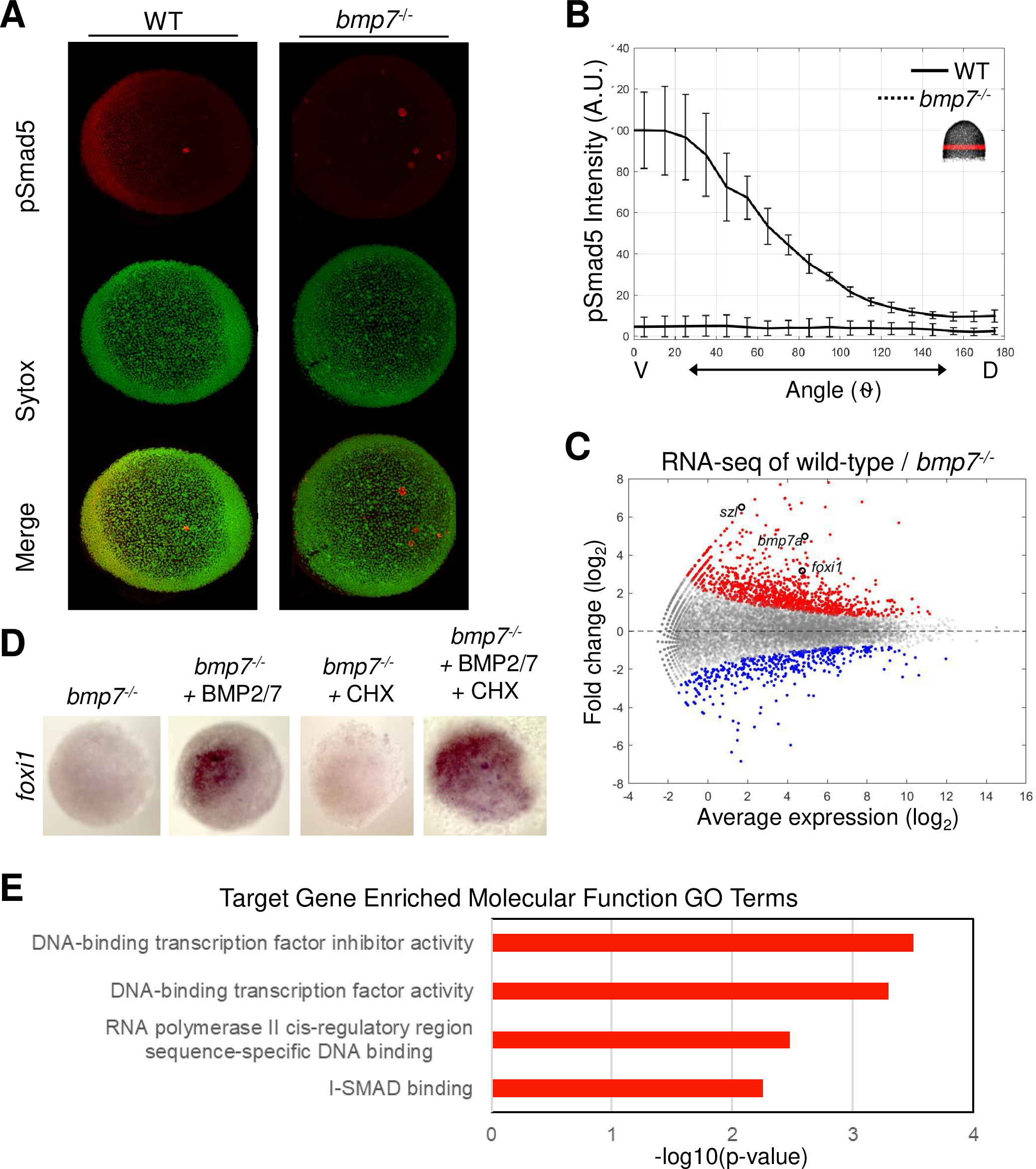

Supplement: S1 Fig — Related to Fig 1. (A) Animal view of the maximum projection of pSmad5 immunofluorescence of a WT and bmp7 mutant mid-gastrula stage (8 hpf) embryo. Nuclei are stained with Sytox Green. (B) Mean pSmad5 profiles of WT (n = 4) (solid line) and bmp7 mutants (n = 3) (dotted line) across the DV axis. Location of the 40-μm band of cells (red) that was averaged is indicated on the embryo in top right corner. See Table D in S1 Data for underlying data. (C) Differential gene expression of WT and bmp7 mutants at early gastrulation (6 hpf) using RNA-seq. Significantly up-regulated genes in WT compared to bmp7 mutants shown in red, and significantly down-regulated genes are shown in blue. All other genes are shown in gray. A subset of known BMP-dependent genes is highlighted. See Table E in S1 Data for underlying data. (D) Animal view of in situ hybridization of foxi1, a known direct target of BMP signaling, in the conditions shown. (E) GO term analysis for molecular functions of the 57 direct target genes. See Table F in S1 Data for underlying data. A.U. is arbitrary units. BMP, Bone Morphogenetic Protein; CHX, cycloheximide; DV, dorsal–ventral; GO, Gene Ontology; hpf, hours post fertilization; pSmad5, phosphorylated Smad5; RNA-seq, RNA sequencing; WT, wild-type. (TIF) [file pbio.3001059.s001.tif]

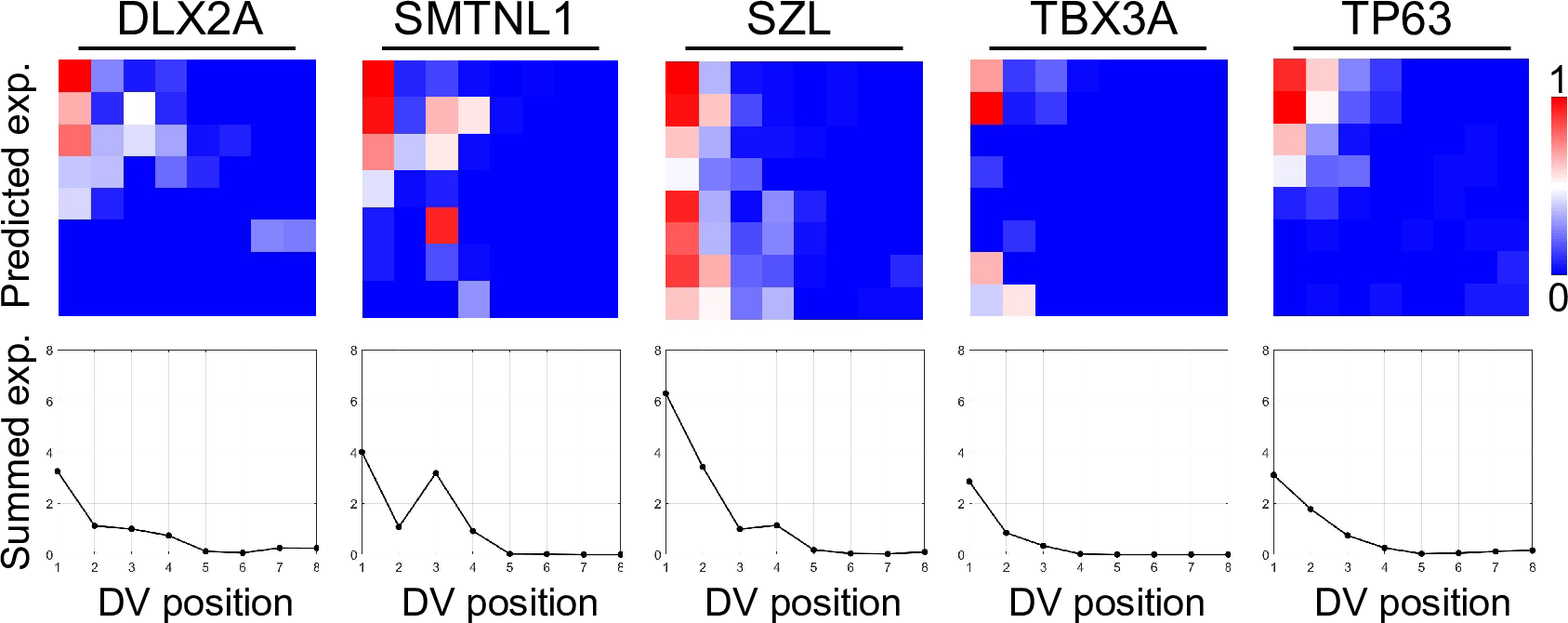

Supplement: S2 Fig — Related to Fig 2. Heat map of gene expression patterns from Seurat analysis for cluster 1 target genes directly up-regulated by BMP signaling and sequenced in scRNA-seq dataset of mid-gastrula (8 hpf) embryos. Cluster 1 target genes are expressed within the first 3 or 4 bins. Predicted expression normalized across all bins. Below are Seurat predicted expression profiles across the DV axis. Each point is the sum of the expression intensity from all bins at 1 DV position. Genes are considered expressed in a bin with greater than 0.5 A.U. of predicted expression. The genes known to expressed dorsally or in the prechordal plate are indicated by asterisks. See Table E in S2 Data for underlying data. A.U. is arbitrary units. BMP, Bone Morphogenetic Protein; DV, dorsal–ventral; hpf, hours post fertilization; scRNA-seq, single-cell RNA sequencing. (TIF) [file pbio.3001059.s002.tif]

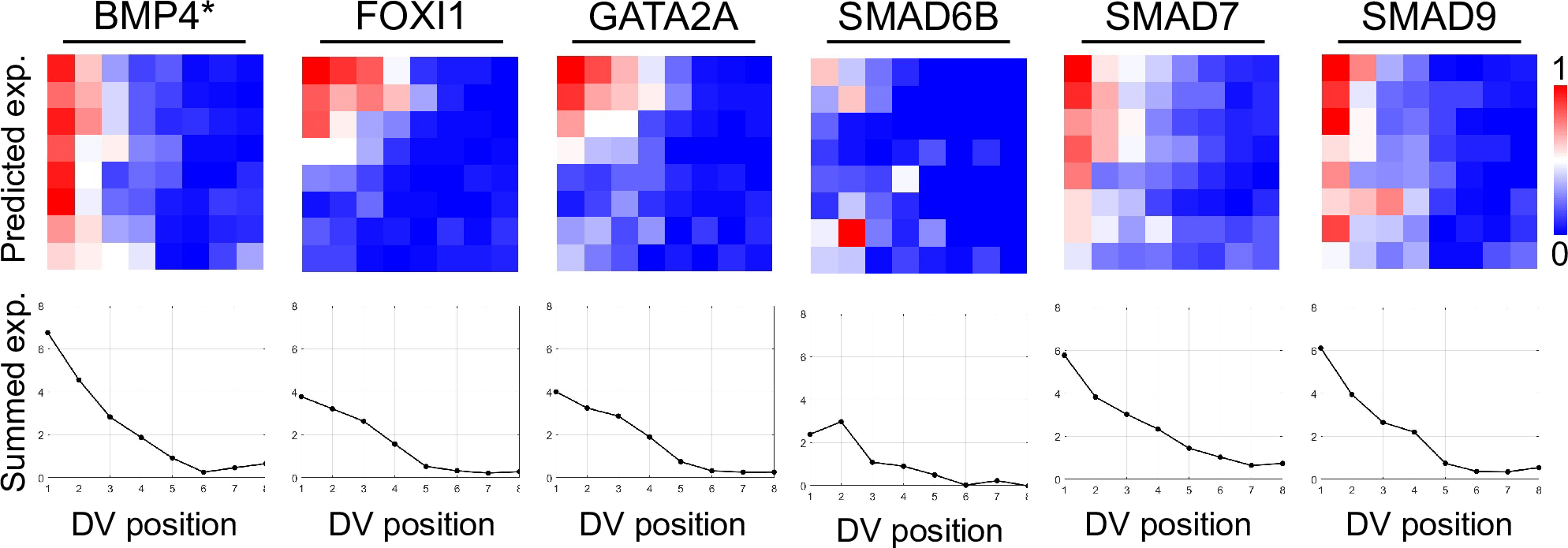

Supplement: S3 Fig — Related to Fig 2. Heat map of gene expression patterns from Seurat analysis for cluster 2 target genes directly up-regulated by BMP signaling and sequenced in scRNA-seq dataset of mid-gastrula (8 hpf) embryos. Cluster 2 target genes are expressed within the first 5 bins. Predicted expression normalized across all bins. Below are Seurat predicted expression profiles across the DV axis. Each point is the sum of the expression intensity from all bins at 1 DV position. Genes are considered expressed in a bin with greater than 0.5 A.U. of predicted expression. The genes known to expressed dorsally or in the prechordal plate are indicated by asterisks. See Table F in S2 Data for underlying data. A.U. is arbitrary units. BMP, Bone Morphogenetic Protein; DV, dorsal–ventral; hpf, hours post fertilization; scRNA-seq, single-cell RNA sequencing. (TIF) [file pbio.3001059.s003.tif]

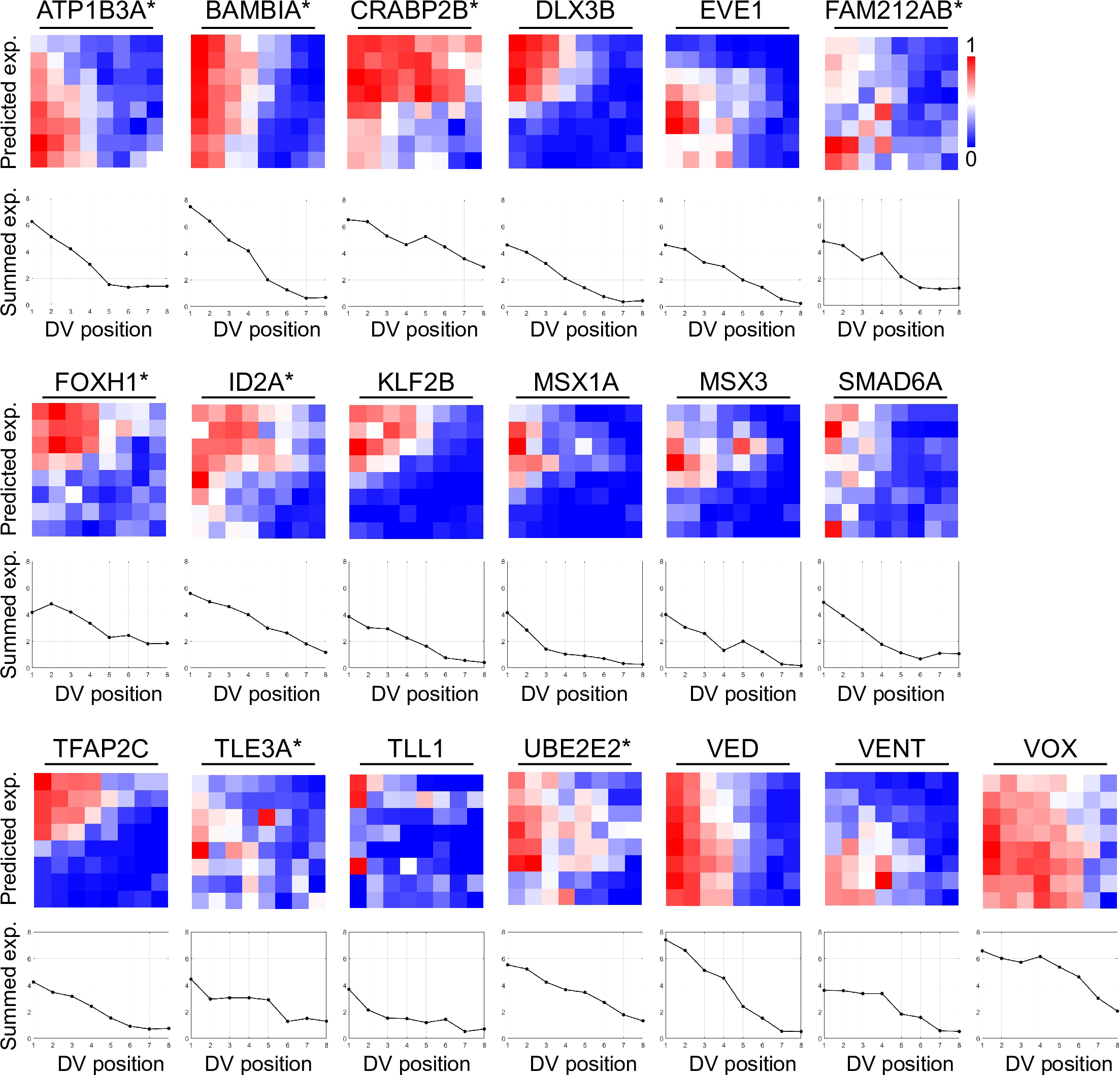

Supplement: S4 Fig — Related to Fig 2. Heat map of gene expression patterns from Seurat analysis for cluster 3 target genes directly up-regulated by BMP signaling and sequenced in scRNA-seq dataset of mid-gastrula (8 hpf) embryos. Cluster 3 target genes are expressed within the first 6 or 7 bins. Predicted expression normalized across all bins. Below are Seurat predicted expression profiles across the DV axis. Each point is the sum of the expression intensity from all bins at 1 DV position. Genes are considered expressed in a bin with greater than 0.5 A.U. of predicted expression. The genes known to expressed dorsally or in the prechordal plate are indicated by asterisks. See Table G in S2 Data for underlying data. A.U. is arbitrary units. BMP, Bone Morphogenetic Protein; DV, dorsal–ventral; hpf, hours post fertilization; scRNA-seq, single-cell RNA sequencing. (TIF) [file pbio.3001059.s004.tif]

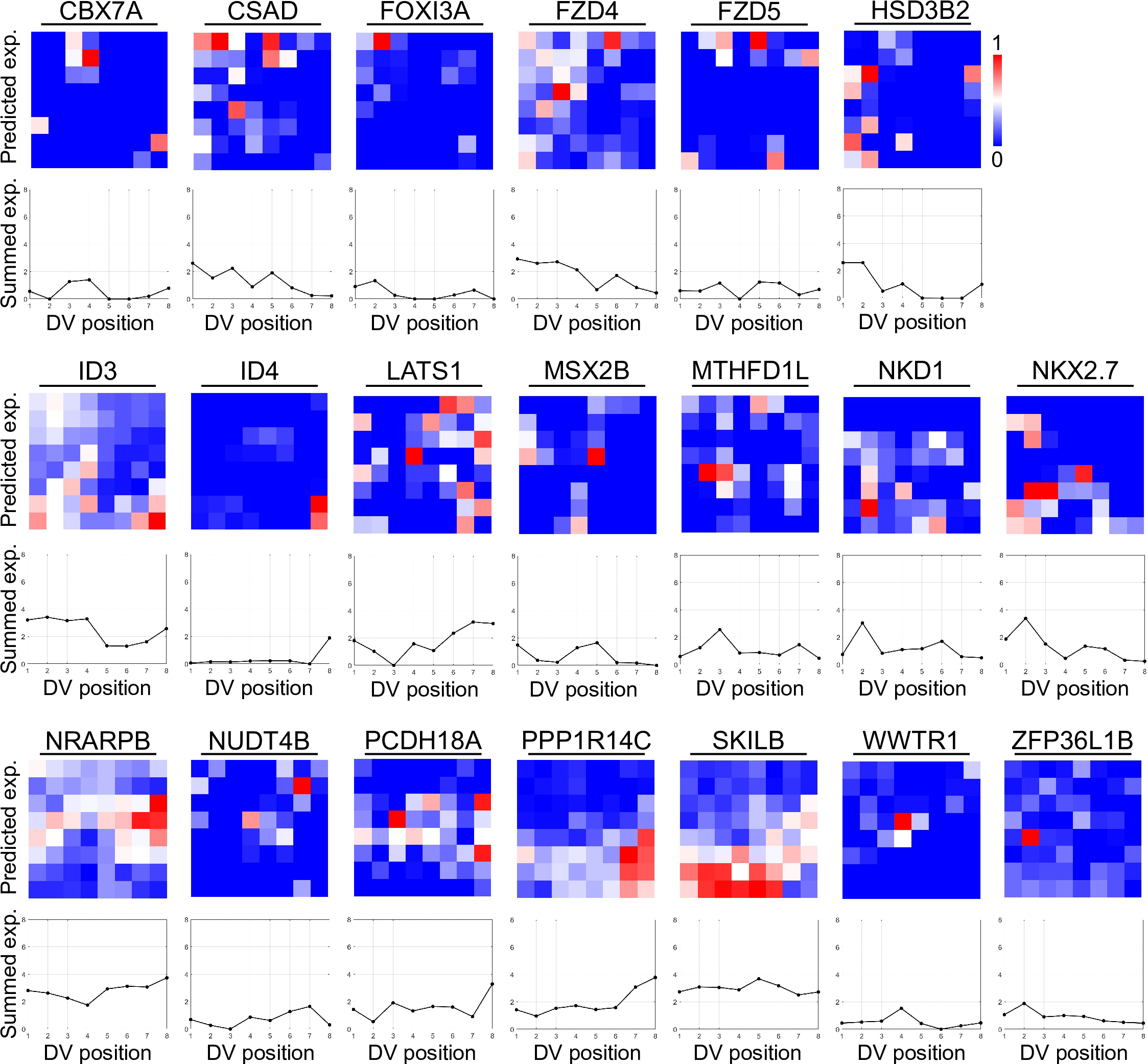

Supplement: S5 Fig — Related to Fig 2. Heat map of gene expression patterns from Seurat analysis for cluster 4 target genes directly up-regulated by BMP signaling and sequenced in scRNA-seq dataset of mid-gastrula (8 hpf) embryos. Cluster 4 target genes have random or uniform expression across the DV axis. Predicted expression normalized across all bins. Below are Seurat predicted expression profiles across the DV axis. Each point is the sum of the expression intensity from all bins at 1 DV position. Genes are considered expressed in a bin with greater than 0.5 A.U. of predicted expression. See Table H in S2 Data for underlying data. A.U. is arbitrary units. BMP, Bone Morphogenetic Protein; DV, dorsal–ventral; hpf, hours post fertilization; scRNA-seq, single-cell RNA sequencing. (TIF) [file pbio.3001059.s005.tif]

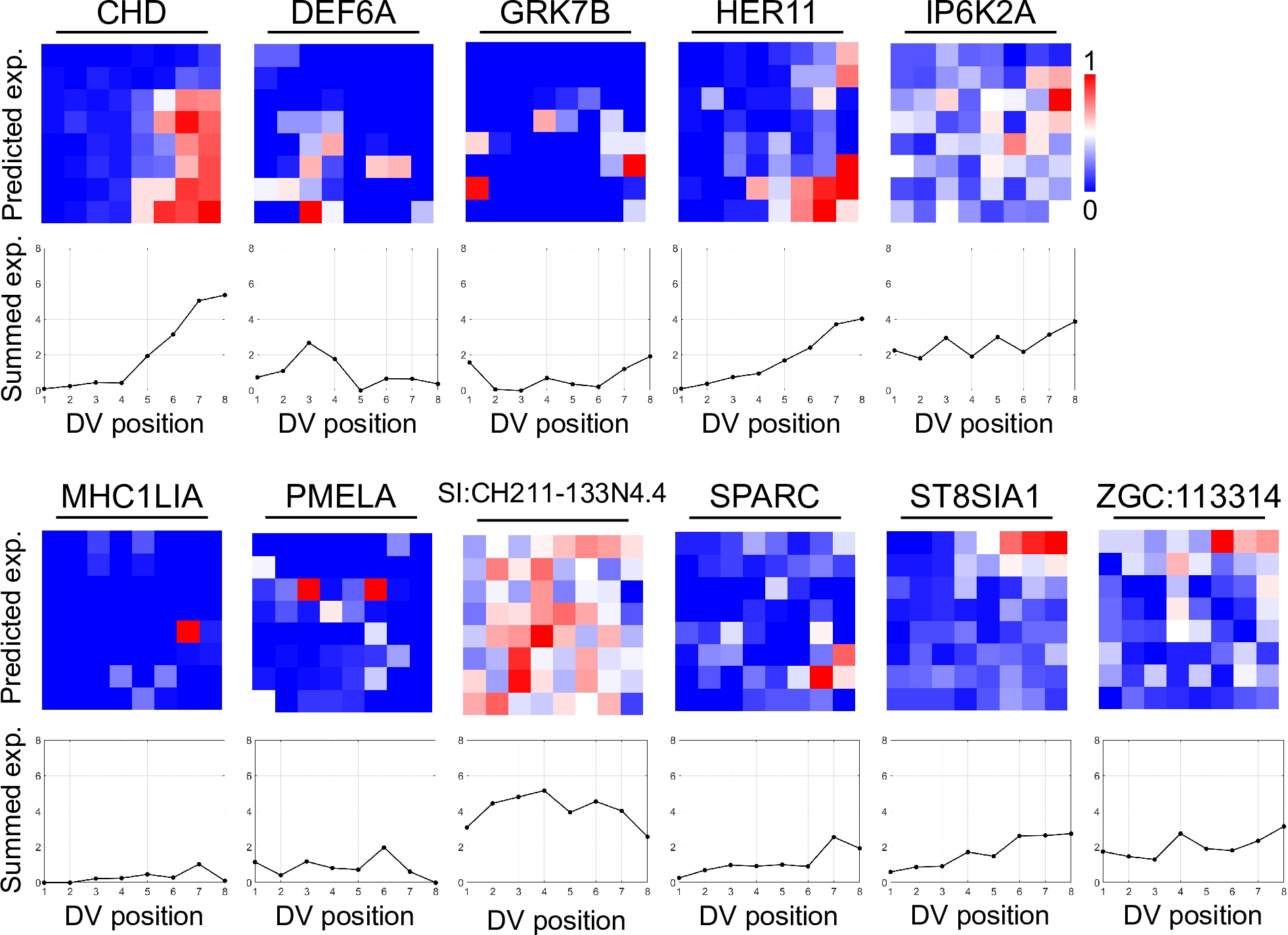

Supplement: S6 Fig — Related to Fig 2. Heat map of gene expression patterns from Seurat analysis for 11 target genes directly down-regulated by BMP signaling that were sequenced in a scRNA-seq dataset of mid-gastrula (8 hpf) embryos. Predicted expression was normalized across all bins. Below are Seurat predicted expression profiles across the DV axis. Each point is the sum of the expression intensity from all bins at 1 DV position. Genes are considered expressed in a bin with greater than 0.5 A.U. of predicted expression. Genes with higher predicted expression in bins 5–8 than bins 1–4 are considered to be dorsally enriched. See Table I in S2 Data for underlying data. A.U. is arbitrary units. BMP, Bone Morphogenetic Protein; DV, dorsal–ventral; hpf, hours post fertilization; scRNA-seq, single-cell RNA sequencing. (TIF) [file pbio.3001059.s006.tif]

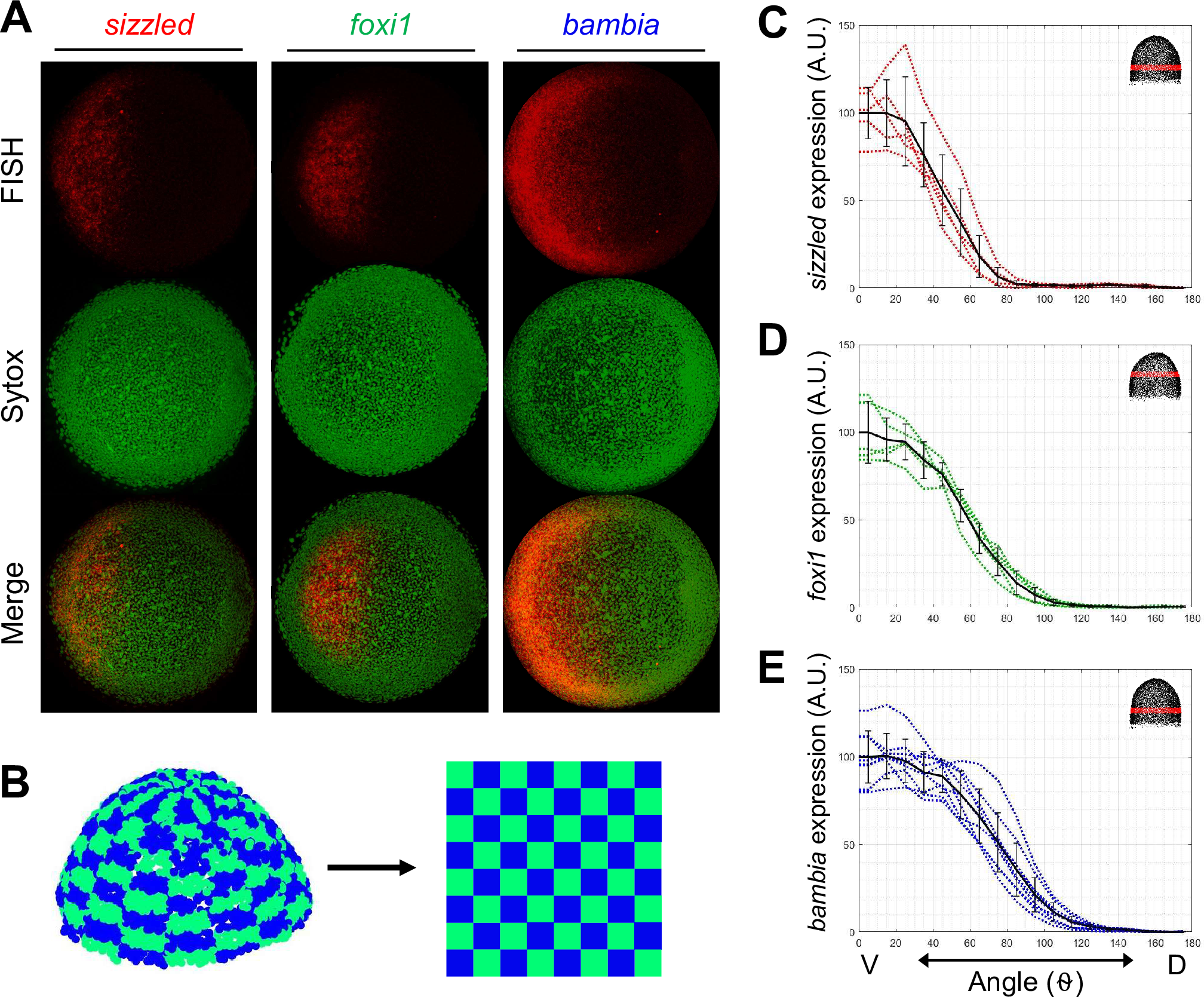

Supplement: S7 Fig — Related to Fig 2. (A) Animal view of a maximum projection of FISH for sizzled, foxi1, and bambia of individual WT embryos at an early gastrula stage (7 hpf). Nuclei are stained with Sytox Green. (B) Schematic of cells of individual embryo partitioned into 128 equally spaced bins. Expression intensity within each bin is averaged. Both halves of the embryo are averaged together into 64 bins, and the expression intensity is normalized across all bins. Expression intensity is displayed as an 8 by 8 heat map. (C–E) Individual (colored) and averaged (black) expression profiles of sizzled (n = 5) (C), foxi1 (n = 5) (D), and bambia (n = 9) (E) across the DV axis of WT embryos at 7 hpf. Location of the 40-μm band of cells that was averaged is indicated on the embryo in the top right corner. The boundary of the expression domain was measured in individual embryos at the position of 10% maximum expression intensity. A.U. is arbitrary units. See Tables J–L in S2 Data for underlying data. DV, dorsal–ventral; FISH, fluorescent in situ hybridization; hpf, hours post fertilization; WT-wild-type. (TIF) [file pbio.3001059.s007.tif]

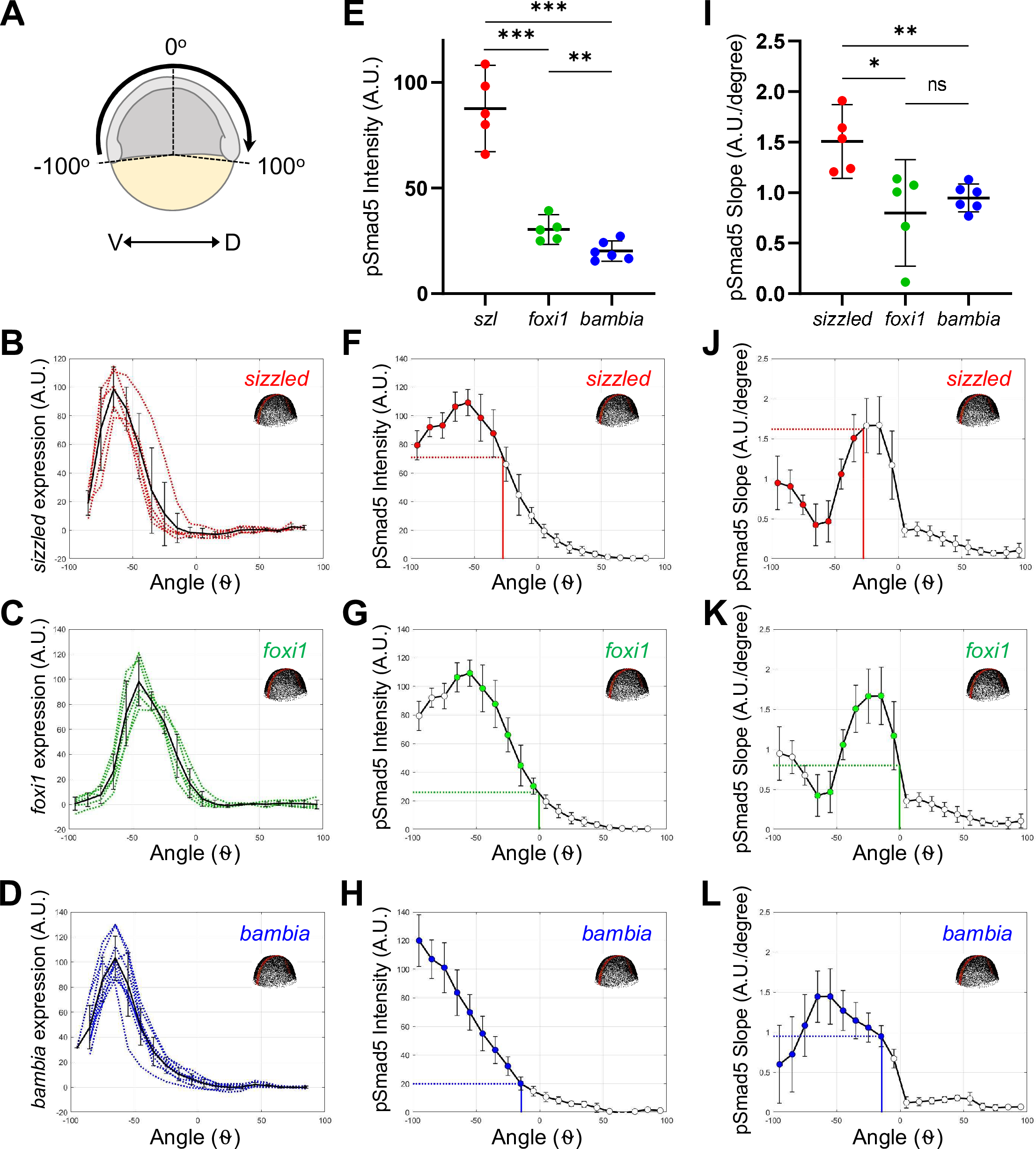

Supplement: S8 Fig — Related to Fig 3. (A) Schematic showing the location where a band of cells over the top of the embryo was averaged, beginning at the ventral margin (−100o) and ending at the dorsal margin (100o). (B–D) Individual (colored) and averaged (black) expression profiles of sizzled (n = 5) (B), foxi1 (n = 5) (C), and bambia (n = 9) (D) across the AV axis of WT embryos at 7 hpf. Location of the 40-μm band of cells that was averaged is indicated in red on the embryo in the top right corner. The boundary of the expression domain was measured in individual embryos at the position of 10% maximum expression intensity. See Tables I–K in S3 Data for underlying data. (E) Measurement of pSmad5 intensity at the location of expression boundaries for sizzled (red), foxi1 (green), and bambia (blue) across the AV axis of WT embryos at 7 hpf. See Table L in S3 Data for underlying data. (F–H) pSmad5 profiles across the AV axis. The intensity is averaged from a 40-μm band of cells around the AV axis at the location shown in red in the right corner embryo schematic of each panel. One WT clutch was used for (F, G) (n = 5), and another clutch was used for (H) (n = 6). Positions of expression boundaries for sizzled (F), foxi1 (G), and bambia (H) are shown as vertical solid lines. Level of pSmad5 at the boundary is indicated as a horizontal dotted line. Colored dots indicate positions where target genes are expressed. See Tables M and N in S3 Data for underlying data. (I) Measurement of pSmad5 slope at the location of expression boundaries for sizzled (red), foxi1 (green), and bambia (blue) across the AV axis of WT embryos at 7 hpf. See Table O in S3 Data for underlying data. (J–L) Slopes of pSmad5 profiles are shown in (F–H). Positions of expression boundaries for sizzled (J), foxi1 (K), and bambia (L) are shown as vertical solid lines. Slope of pSmad5 at the boundary is indicated as a horizontal dotted line. Colored dots indicate positions where target genes are expressed. See Tables P and Q in [file pbio.3001059.s008.tif]

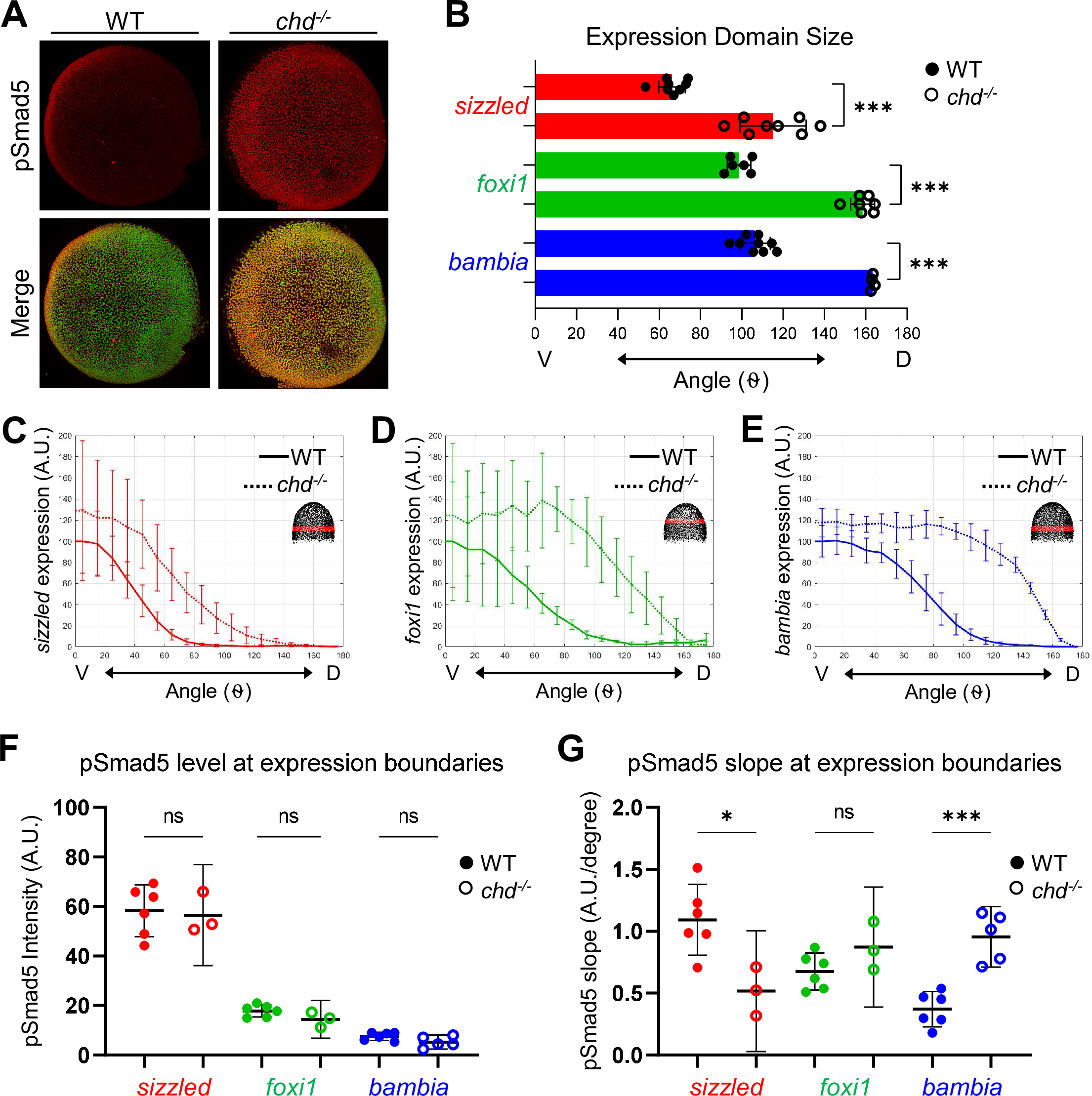

Supplement: S9 Fig — Related to Fig 4. (A) Animal view of the maximum projection of pSmad5 immunofluorescence in an individual WT and chordin mutant at an early gastrula stage (7 hpf). Merged image with Sytox Green staining nuclei. Because the chordin mutant displays high pSmad5 levels throughout the embryo, a lower confocal laser intensity gain was used in imaging the WT and chordin mutant embryos in this experiment compared to other pSmad5 imaging experiments. (B) Position of expression boundaries for sizzled (red), foxi1 (green) and bambia (blue) in individual WT (filled) and chordin mutant (opened) embryos. See Table G in S4 Data for underlying data. (C–E) Average expression profiles of sizzled (C), foxi1 (D), and bambia (E) across the DV axis of WT (solid line) and chordin mutant (dotted line) embryos. Location of the 40-μm band of cells that was averaged is indicated on the embryo in the top right corner. See Tables H–J in S4 Data for underlying data. (F) Measurement of pSmad5 intensity at the location of expression boundaries for sizzled (red), foxi1 (green), and bambia (blue) across the DV axis of WT (filled) and chordin mutant (opened) embryos at 7 hpf. See Table K in S4 Data for underlying data. (G) Measurement of pSmad5 gradient slope at the location of expression boundaries for sizzled (red), foxi1 (green), and bambia (blue) across the DV axis of WT (filled) and chordin mutant (opened) embryos at 7 hpf. See Table L in S4 Data for underlying data. *P < 0.05, ***P < 0.001 in comparing DV position, pSmad5 levels, and pSmad5 slopes using unpaired 2-tailed Student t tests. NS is not significant. A.U. is arbitrary units. DV, dorsal–ventral; hpf, hours post fertilization; pSmad5, phosphorylated Smad5; WT, wild-type. (TIF) [file pbio.3001059.s009.tif]

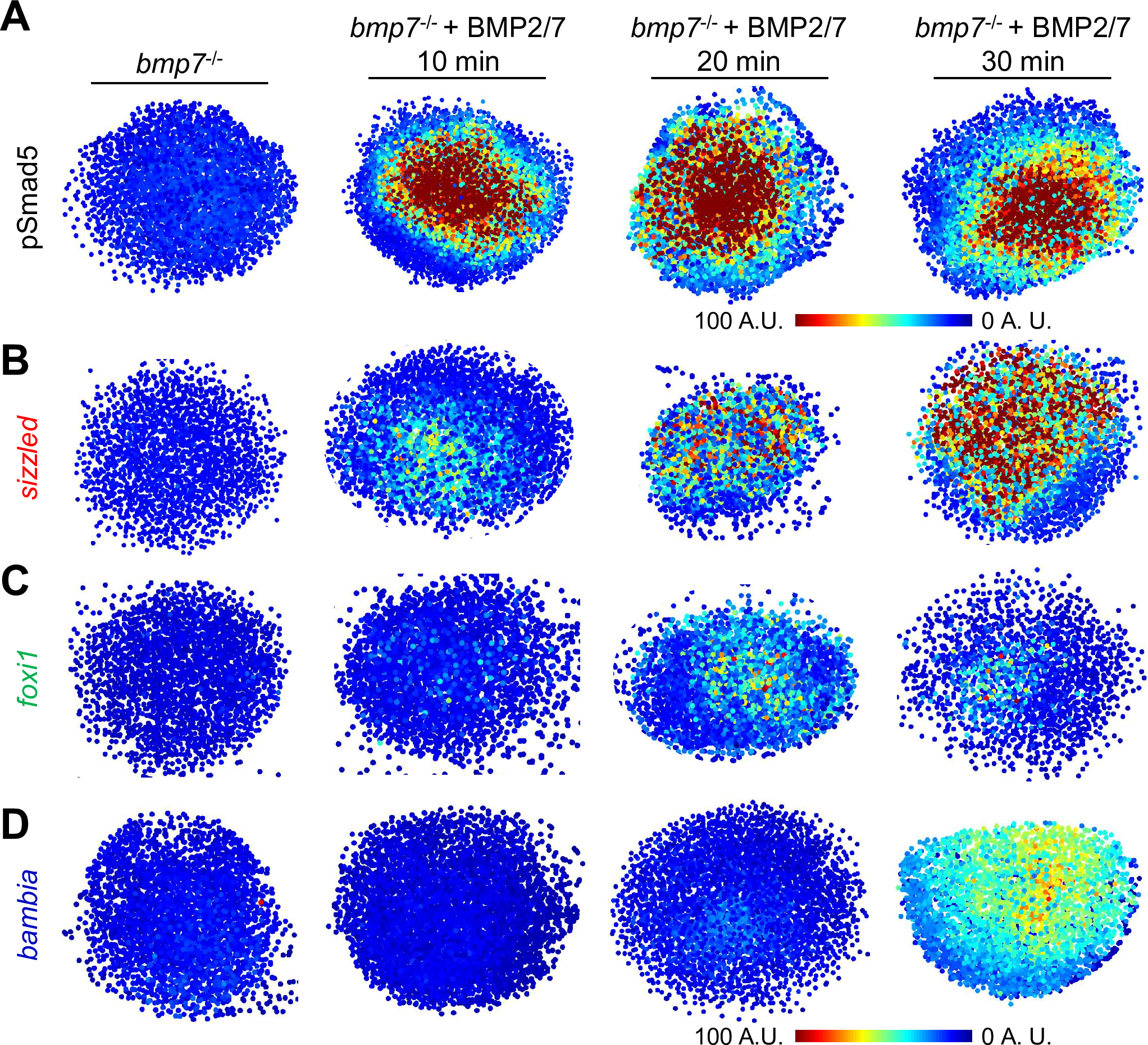

Supplement: S10 Fig — Related to Fig 5. (A) Representative immunostaining of pSmad5 intensities of an uninjected bmp7 mutant (n = 10) and bmp7 mutants injected with 5 pg of BMP2/7 protein and fixed after 10, 20, and 30 minutes after injection (n = 10, n = 10, n = 10). Animal pole is facing up. (B–D) Representative FISH for sizzled (E) (n = 10, n = 11, n = 10, n = 10), foxi1 (F) (n = 5, n = 5, n = 5, n = 5), and bambia (G) (n = 5, n = 5, n = 5, n = 5) in uninjected bmp7 mutants and bmp7 mutants injected with 5 pg of BMP2/7 protein and fixed 10, 20, and 30 minutes after injection. Animal pole is facing up. A.U. is arbitrary units. BMP, Bone Morphogenetic Protein; FISH, fluorescent in situ hybridization; pSmad5, phosphorylated Smad5. (TIF) [file pbio.3001059.s010.tif]

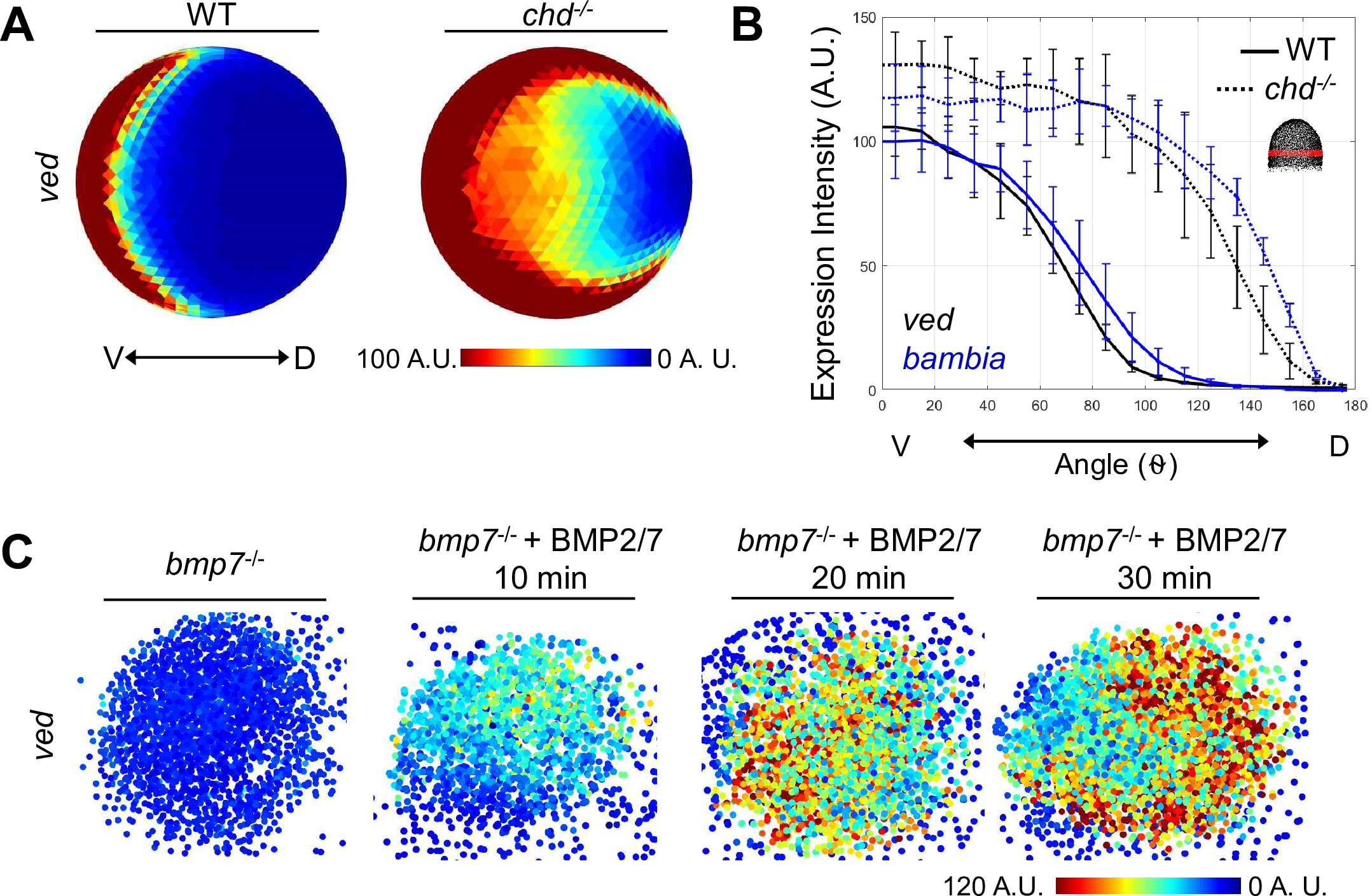

Supplement: S11 Fig — Related to Fig 5. (A) Animal views of average FISH signal of ved in WT embryos (n = 6) and chordin mutants (n = 5) at an early gastrula stage (7 hpf). (B) Average expression profiles of ved (black) and bambia (blue) in WT (solid line) and chordin mutants (dotted line). Location of 40-μm band of cells that was averaged is indicated on embryo in right corner. See Table A in S5 Data for underlying data. (C) Representative FISH for ved in bmp7 mutants uninjected or injected with 5 pg of BMP2/7 protein and fixed 10, 20, and 30 minutes after injection (n = 5, n = 5, n = 5, n = 5). Animal pole is facing up. A.U. is arbitrary units. BMP, Bone Morphogenetic Protein; FISH, fluorescent in situ hybridization; hpf, hours post fertilization; WT, wild-type. (TIF) [file pbio.3001059.s011.tif]

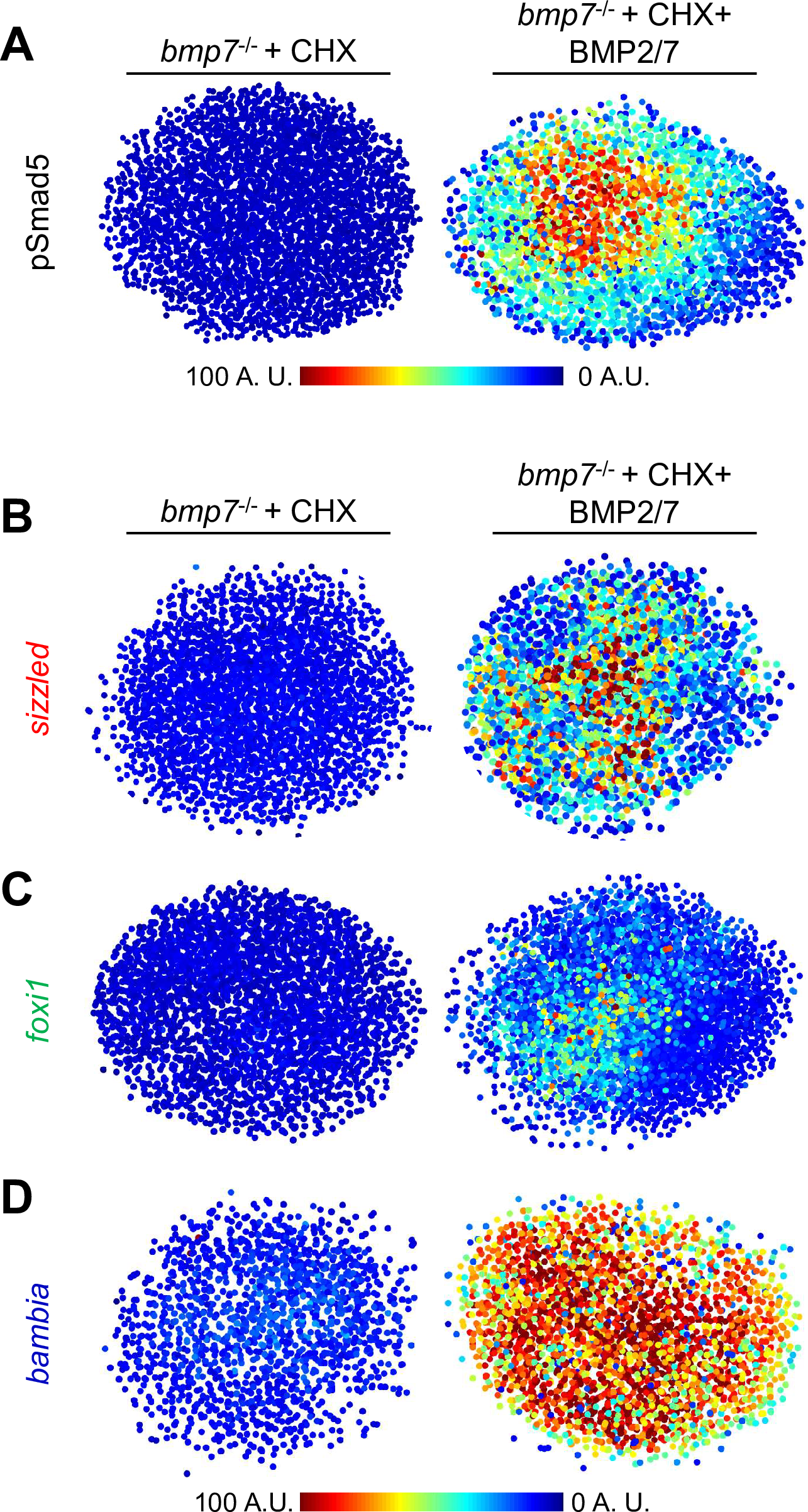

Supplement: S12 Fig — Related to Fig 5. (A) Representative immunostaining of pSmad5 intensities of an uninjected bmp7 mutant treated with CHX (n = 15) and a bmp7 mutant treated with CHX and then injected with 5 pg of BMP2/7 protein (n = 15). Animal pole is facing up. (B–D) Representative FISH in CHX-treated bmp7 mutants that were either uninjected or injected with 5 pg of BMP2/7 protein for sizzled (E) (n = 10 uninjected, n = 11 injected), foxi1 (F) (n = 10, n = 10), and bambia (G) (n = 10, n = 11). Animal pole is facing up. A.U. is arbitrary units. BMP, Bone Morphogenetic Protein; CHX, cycloheximide; FISH, fluorescent in situ hybridization; pSmad5, phosphorylated Smad5. (TIF) [file pbio.3001059.s012.tif]

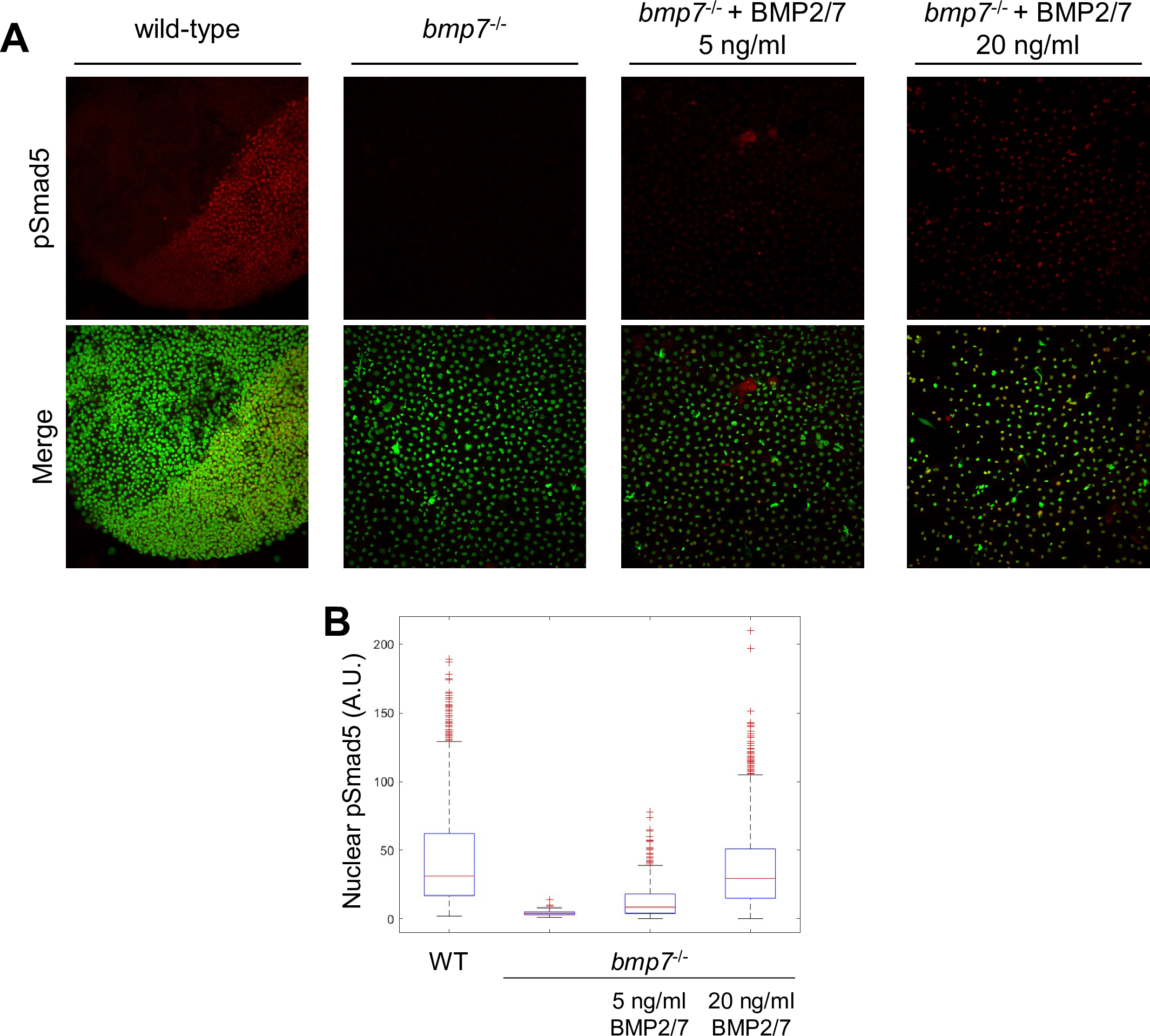

Supplement: S13 Fig — Related to Fig 6. (A) Representative maximum projection of pSmad5 immunofluorescence in whole-mount WT embryo, disassociated cells from bmp7 mutants and disassociated cells from bmp7 mutants treated with 5 or 20 ng/ml BMP2/7 protein. Merged image with Sytox Green stained nuclei. (B) Quantification of nuclear pSmad5 intensities of individual cells in whole-mount WT embryos, disassociated cells from bmp7 mutants and disassociated cells from bmp7 mutants treated with 5 or 20 ng/ml BMP2/7 protein. See Table A in S6 Data for underlying data. A.U. is arbitrary units. BMP, Bone Morphogenetic Protein; pSmad5, phosphorylated Smad5; WT, wild-type. (TIF) [file pbio.3001059.s013.tif]

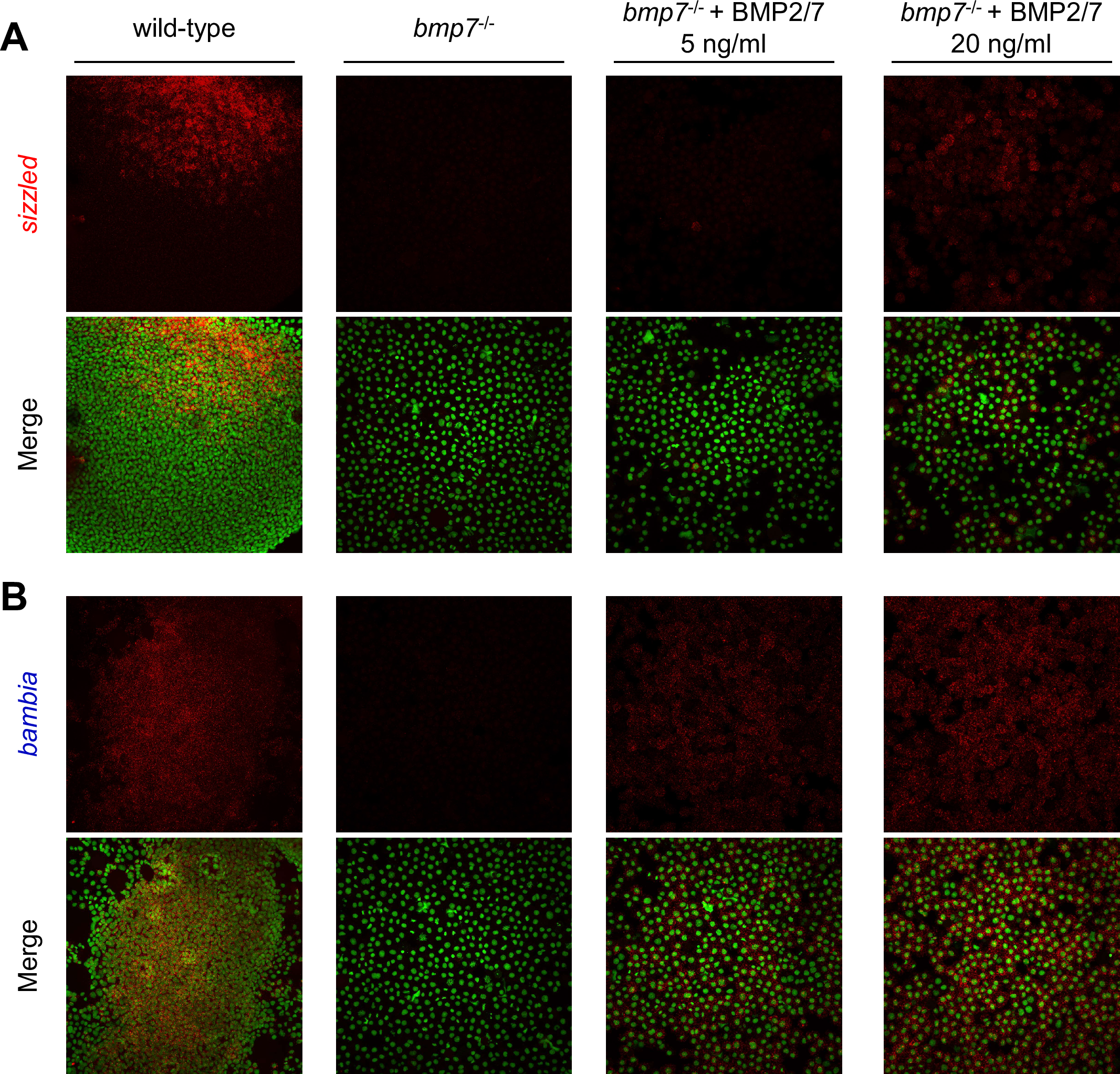

Supplement: S14 Fig — Related to Fig 6. (A) Representative maximum projection of sizzled FISH in whole-mount WT embryos, disassociated cells from bmp7 mutants and disassociated cells from bmp7 mutants treated with 5 or 20 ng/ml BMP2/7 protein. Merged image with Sytox Green stained nuclei. (B) Representative maximum projection of bambia FISH in whole-mount WT embryos, disassociated cells from bmp7 mutants and disassociated cells from bmp7 mutants treated with 5 or 20 ng/ml BMP2/7 protein. Merged image with Sytox Green stained nuclei. BMP, Bone Morphogenetic Protein; FISH, fluorescent in situ hybridization; WT, wild-type. (TIF) [file pbio.3001059.s014.tif]
