# Supplementary material for: The BMP signaling gradient is interpreted through concentration thresholds in dorsal–ventral axial patterning
Source: PLoS Biol. 2021 Jan 22;19(1):e3001059. doi: 10.1371/journal.pbio.3001059 (PMC7857602; doi:10.1371/journal.pbio.3001059)
Supplement: S1 Table — Related to Fig 1 and S1 Fig. List of names and RefSeq accession numbers of genes directly activated by BMP signaling during gastrulation, as well as the corresponding cluster number based on the Seurat predicted expression profile. Clusters 1, 2, and 3 are genes with predicted ventrally enriched expression. Cluster 4 contains the genes with predicted uniform profiles or significant dorsal expression. NS indicates genes that were not sequenced in the Farrell et al. (2018) scRNA-seq dataset. BMP, Bone Morphogenetic Protein; scRNA-seq, single-cell RNA sequencing. (DOCX) [file pbio.3001059.s015.docx]

Supplemental Table 1. BMP upregulated target genes

Related to Figure 1 and S1.

List of names and RefSeq accession numbers of genes directly activated by BMP signaling during gastrulation, as well as the corresponding cluster number based on the Seurat predicted expression profile. Clusters 1, 2, 3 are genes with predicted ventrally enriched expression. Cluster 4 contains the genes with predicted uniform profiles or significant dorsal expression. NS indicates genes that were not sequenced in the Farrell et al., 2018 scRNA-seq dataset.

| **Gene** | **Transcript** | **Cluster #** |
| --- | --- | --- |
| *dlx2a* | NM_131311 | 1 |
| *smtnl1* | NM_001013321 | 1 |
| *szl* | NM_181663 | 1 |
| *tbx3a* | NM_001101670 | 1 |
| *tp63* | NM_152987 | 1 |
| *bmp4* | NM_131342 | 2 |
| *foxi1* | NM_181735 | 2 |
| *gata2a* | NM_131233 | 2 |
| *smad6b* | NM_001045051 | 2 |
| *smad7* | NM_175082 | 2 |
| *smad9* | NM_001328499 | 2 |
| *atp1b3a* | NM_131221 | 3 |
| *bambia* | NM_131784 | 3 |
| *crabp2b* | NM_001320394 | 3 |
| *dlx3b* | NM_131322 | 3 |
| *eve1* | NM_131114 | 3 |
| *fam212ab* | NM_199788 | 3 |
| *foxh1* | NM_131502 | 3 |
| *id2a* | NM_201291 | 3 |
| *klf2b* | NM_131857 | 3 |
| *msx1a* | NM_131273 | 3 |
| *msx3* | NM_131272 | 3 |
| *smad6a* | NM_001024810 | 3 |
| *tfap2c* | NM_001008576 | 3 |
| *tle3a* | NM_131012 | 3 |
| *tll1* | NM_131010 | 3 |
| *ube2e2* | NM_001003494 | 3 |
| *ved* | NM_183074 | 3 |
| *vent* | NM_131700 | 3 |
| *vox* | NM_131698 | 3 |
| *cbx7a* | NM_001017853 | 4 |
| *csad* | NM_001007348 | 4 |
| *foxi3a* | NM_198917 | 4 |
| *fzd4* | NM_001305469 | 4 |
| *fzd5* | NM_131134 | 4 |
| *hsd3b2* | NM_212797 | 4 |
| *id3* | NM_152967 | 4 |
| *id4* | NM_001039990 | 4 |
| *lats1* | NM_001020510 | 4 |
| *msx2b* | NM_131276 | 4 |
| *mthfd1l* | NM_001242996 | 4 |
| *nkd1* | NM_001043333 | 4 |
| *nkx2.7* | NM_131419 | 4 |
| *nrarpb* | NM_181496 | 4 |
| *nudt4b* | NM_001004648 | 4 |
| *pcdh18a* | NM_001115058 | 4 |
| *ppp1r14c* | NM_001029963 | 4 |
| *skilb* | NM_001130669 | 4 |
| *wwtr1* | NM_001037696 | 4 |
| *zfp36l1b* | NM_199649 | 4 |
| *cdx1a* | NM_212836 | NS |
| *dram1* | NM_001006049 | NS |
| *kcnh6a* | NM_212837 | NS |
| *neu4* | NM_001020548 | NS |
| *si:ch211-107n13.1* | NM_001082926 | NS |
| *slc14a2* | NM_001020519 | NS |
| *tdrd7b* | NM_001353929 | NS |

**REFERENCES**

Farrell JA, Wang Y, Riesenfeld SJ, Shekhar K, Regev A, Schier AF. Single-cell reconstruction of developmental trajectories during zebrafish embryogenesis. Science (New York, NY). 2018;360(6392).
