# Supplementary material for: The BMP signaling gradient is interpreted through concentration thresholds in dorsal–ventral axial patterning
Source: PLoS Biol. 2021 Jan 22;19(1):e3001059. doi: 10.1371/journal.pbio.3001059 (PMC7857602; doi:10.1371/journal.pbio.3001059)
Supplement: S2 Table — Related to S6 Fig. List of names and RefSeq accession numbers of genes directly inhibited by BMP signaling during gastrulation, as well as the expression domain predicted by Seurat. NE indicates predicted expression is not enriched along the DV axis. NS indicates genes that were not sequenced in the Farrell et al. (2018) scRNA-seq dataset. BMP, Bone Morphogenetic Protein; DV, dorsal–ventral; scRNA-seq, single-cell RNA sequencing. (DOCX) [file pbio.3001059.s016.docx]

Supplemental Table 2. BMP downregulated target genes

Related to Figure S3.

List of names and RefSeq accession numbers of genes directly inhibited by BMP signaling during gastrulation, as well as the expression domain predicted by Seurat. NE indicates predicted expression is not enriched along the DV axis. NS indicates genes that were not sequenced in the Farrell et al., 2018 scRNA-seq dataset.

| **Gene** | **Transcript** | **Predicted Domain** |
| --- | --- | --- |
| *chrd* | NM_130973 | dorsal |
| *her11* | NM_001003886 | dorsal |
| *sparc* | NM_001001942 | dorsal |
| *st8sia1* | NM_001327841 | dorsal |
| *zgc:113314* | NM_001033753 | dorsal |
| *def6a* | NM_201040 | NE |
| *grk7b* | NM_001033090 | NE |
| *ip6k2a* | NM_201470 | NE |
| *mhc1lia* | NM_001327882 | NE |
| *pmela* | NM_001045330 | NE |
| *si:ch211-133n4.4* | NM_001077376 | NE |
| *cpa4* | NM_001002217 | NS |
| *cyp2p9* | NM_200620 | NS |
| *zgc:86896* | NM_001002100 | NS |

**REFERENCES**

Farrell JA, Wang Y, Riesenfeld SJ, Shekhar K, Regev A, Schier AF. Single-cell reconstruction of developmental trajectories during zebrafish embryogenesis. Science (New York, NY). 2018;360(6392).
